# Dysregulated LRRK2 Signaling in Response to Endoplasmic Reticulum Stress Leads to Dopaminergic Neuron Degeneration in *C. elegans*

**DOI:** 10.1371/journal.pone.0022354

**Published:** 2011-08-03

**Authors:** Yiyuan Yuan, Pengxiu Cao, Mark A. Smith, Kristopher Kramp, Ying Huang, Naoki Hisamoto, Kunihiro Matsumoto, Maria Hatzoglou, Hui Jin, Zhaoyang Feng

**Affiliations:** 1 Department of Pharmacology, School of Medicine, Case Western Reserve University, Cleveland, Ohio, United States of America; 2 Department of Pathology, School of Medicine, Case Western Reserve University, Cleveland, Ohio, United States of America; 3 Department of Nutrition, School of Medicine, Case Western Reserve University, Cleveland, Ohio, United States of America; 4 Department of Molecular Biology, Graduate School of Science, Institute for Advanced Research, Nagoya Solution-Oriented Research for Science and Technology, Japan Science and Technology Corporation, Chikusa-ku, Nagoya, Japan; 5 Department of Physiology, College of Medicine, Xi'an Jiaotong University, Xi'an, Shaanxi, China; Brown University, United States of America

## Abstract

Mutation of leucine-rich repeat kinase 2 (LRRK2) is the leading genetic cause of Parkinson's Disease (PD), manifested as age-dependent dopaminergic neurodegeneration, but the underlying molecular mechanisms remain unclear. Multiple roles of LRRK2 may contribute to dopaminergic neurodegeneration. Endoplasmic reticulum (ER) stress has also been linked to PD pathogenesis, but its interactive mechanism with PD genetic factors is largely unknown. Here, we used *C. elegans*, human neuroblastoma cells and murine cortical neurons to determine the role of LRRK2 in maintaining dopaminergic neuron viability. We found that LRRK2 acts to protect neuroblastoma cells and *C. elegans* dopaminergic neurons from the toxicity of 6-hydroxydopamine and/or human α-synuclein, possibly through the p38 pathway, by supporting upregulation of GRP78, a key cell survival molecule during ER stress. A pathogenic LRRK2 mutant (G2019S), however, caused chronic p38 activation that led to death of murine neurons and age-related dopaminergic-specific neurodegeneration in nematodes. These observations establish a critical functional link between LRRK2 and ER stress.

## Introduction

Parkinson's disease (PD) is a major neurodegenerative disease that results from the loss of dopaminergic (DAergic) neurons in the *substantia nigra* of patients. The leading genetic cause of PD is mutation of leucine-rich repeat kinase 2 (LRRK2) [1], [2], which is associated with both familial and idiopathic PD [3], [4], and represents a potential therapeutic target [5]. The biological functions of LRRK2 remain poorly defined and the molecular mechanisms by which LRRK2 pathogenic mutations contribute to neurodegeneration are largely unknown [6], [7]. Transgenic animal models featuring wild type (WT) and mutant forms of human LRRK2 have been generated in nematodes [8], [9], flies [10]–[12] and rodents [13]–[15]. In these models, LRRK2 was found to interact with components involved in the autophagy-lysosomal pathway [16] or protein quality control [15], [17], modulate oxidative stress [8], [17], regulate protein synthesis [18] and mediate the microRNA pathway [19], indicating that multiple mechanisms may underlie LRRK2 pathology [7], [19]. Moreover, confusing and even conflicting experimental results have been reported. For example, observations made in various animal models with gain- or loss- of LRRK2 kinase activity have led to conclusions that LRRK2 kinase activity is protective, deleterious or dispensable for neuronal survival [8], [10], [20]–[22]. Therefore, it is important to precisely define the signaling pathways of LRRK2 and their distinct contribution to DAergic neuron viability.

Mutations of human α-synuclein (hαSyn) or exposure to neurotoxins such as 6-hydroxydopamine (6-OHDA) also causes DAergic neuron degeneration in humans and animal PD models [23]–[27]. Recently, a pathophysiological interplay between LRRK2 and α-synuclein was demonstrated by experiments in which overexpression of LRRK2 enhanced pathogenic α-synuclein-induced neurophathological abnormalities in transgenic mice [28]. The molecular mechanism(s) underlying this important observation and other reported interactions between PD genetic/environmental factors remain unclear, however.

The nematode, *C. elegans* may constitute a useful model to study genetic mechanisms underlying its pathology. For example, nematodes were used to study the function of LRK-1, the sole nematode homolog of LRRK2, in synaptic protein sorting [29] and organism survival after exposure to mitochondrial toxins [8]. But, the role of LRRK2 kinase activity in maintaining DAergic neuron viability has not been studied in nematodes, although expression of human pathogenic LRRK2 in nematodes leads to DAergic neuron degeneration and motor activity deficit [30].

In the work reported here, we investigated the molecular mechanism by which LRRK2 impacts the viability of DAergic neurons of *C. elegans*, a human neuroblastoma cell line and murine cortical neurons. We found that deficient LRRK2 signaling increased hαSyn- or 6-OHDA-mediated neuroblastoma cell death or nematode DAergic neuron degeneration. LRRK2 executes this cytoprotective role by supporting synthesis of GRP78, a chaperone that plays a key role in promoting cell survival following ER stress [31], possibly signaling through the p38 pathway. On the other hand, human pathogenic G2019S mutant LRRK2, which exhibits enhanced kinase activity and causes chronic p38 activation in human cells, displayed adult-onset, progressive, DAergic-specific neurodegeneration that was dependent on functional p38. Overactive LRRK2 signaling was also found to promote death of G2019S LRRK2-expressing primary murine cortical neurons through p38. These data suggest that “appropriate” LRRK2 activity in neurons is cytoprotective, whereas overactive LRRK2 is degenerative. Interestingly, this mechanism of LRRK2 is conserved between nematodes and human cell lines. Together, we identified an unexpected functional link between LRRK2 signaling and ER stress response.

## Results

### The DAergic neurotoxicity of 6-OHDA is potentiated in *lrk-1* mutant nematodes

To investigate the molecular mechanism by which LRRK2 impacts neuron viability, we chose to use the DAergic neurotoxin, 6-OHDA, for its experimental convenience, and nematodes for their readily manipulated genetics. 6-OHDA has been used previously in mammals [32] and *C. elegans* [25] to induce DAergic neuron degeneration and study PD pathology. In nematode hermaphrodites, 6-OHDA-induced DAergic neuron degeneration was dose-dependent and required a functional dopamine transporter (DAT) [25]. Degeneration of these cells in nematode hermaphrodites following either 6-OHDA treatment or hαSyn expression was evidenced by Electron Microscopy (EM) or tyrosine hydroxylase (TH) immuonstaining. Degeneration was also visualized *in vivo* through DAergic neuron-specific expression of the fluorescent markers, GFP or DsRed [25], [33]. Using this method, we observed dose-dependent 6-OHDA-induced DAergic neuron degeneration in our DsRed expressing nematode line and found that this degeneration could be prevented by co-treatment with the DAT blocker, imipramine (Figure S1). Nematode hermaphrodites have a total of eight DAergic neurons: 4 CEPs, 2 ADEs and 2 PDEs. All of these DAergic neurons showed similar 6-OHDA-induced, imipramine-blockable degeneration, although only the DAergic neurons located in the nematode head (CEPs and ADEs) are shown in Figure S1A–F.

**Figure 1:**
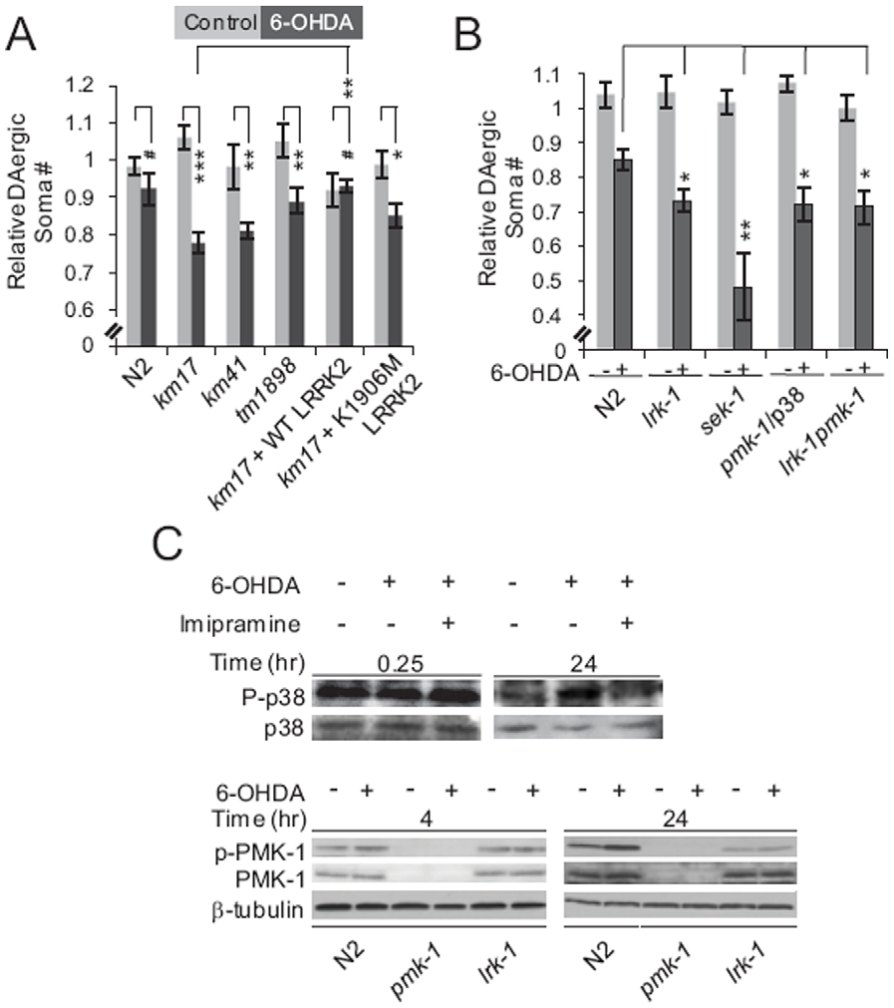
LRK-1 functions upstream of p38 to protect against 6-OHDA-induced DAergic neuron degeneration in *C. elegans*. (**A**) Human LRRK2 can functionally substitute for LRK-1 to protect against 6-OHDA-induced DAergic neuron degeneration. Day 4 nematodes of the indicated genotypes (N2, three different *lrk-1* loss-of-function mutants (*km17*, *km41* and *tm1898*), and *km17* mutants expressing human WT or K1906M (kinase inactive) LRRK2 in their DAergic neurons) were treated with vehicle (grey bars) or 2 mM 6-OHDA (black bars) for 1 h at L3. DAergic neurons were counted and normalized as described in Experimental Procedures. Data represent the mean ± SEM of three independent experiments. Each experiment employed 20–30 nematodes. ^#^, p>0.05, *, p<0.05, **, p<0.01, and ***, p<0.005 by t-test. (**B**) *lrk-1*, *pmk-1* and *sek-1* function in the same genetic pathway to protect against 6-OHDA-induced DAergic neurodegeneration. Day 4 nematodes of the indicated genotypes (N2, *km17* mutant (*lrk-1*), *pmk-1*/p38 *null* (*pmk-1*/p38), *lrk-1pmk-1* double *null* (*lrk-1*/*pmk-1*) and *sek-1*/MKK6 *null* (*sek-1*)) were treated with vehicle (light grey bars) or 2 mM 6-OHDA (dark grey bars) for 1 h at L3. DAergic neurons were counted and normalized as described in Experimental Procedures. Data represent the mean ± SEM of four independent experiments. Each experiment employed 20–30 nematodes. *, p<0.05 and **, p<0.01 by one-way ANOVA with Dunnett's test. (**C**) 6-OHDA treatment induces p38 phosphorylation in nematodes (upper panel). Nematodes were treated with 2 mM 6-OHDA and 1 mM imipramine for 1 h at L3, and harvested after 15 mins or 24 hrs as indicated above each lane. Western blots were probed with antibodies specific to phosphorylated p38 (P-p38) or total p38. 6-OHDA-mediated induction of p38 phosphorylation in nematodes requires LRK-1 activity (lower panel). N2, *pmk-1* mutant or *lrk-1* mutant (*km17*) nematodes were exposed to 2 mM 6-OHDA for 1 hour at L3 and harvested after 4 hrs or 24 hrs. No p38 band was detected by Western blot in lysates from *pmk-1*/p38 *null* mutant animals.

To test whether LRK-1, the sole nematode homolog of LRRK2 [29], plays a role in maintaining DAergic neuron viability, we examined the effect of 6-OHDA treatment on *lrk-1* mutant nematodes as compared to wild type (Bristol N2) nematodes. We found that a concentration of 6-OHDA (2 mM) that produced little or no DAergic neuron degeneration in wild type nematodes (Figure 1A and Figure S1), induced substantially more severe DAergic neuron degeneration in several nematode strains with loss-of-function *lrk-1* mutations (Figure 1A and Figure S1G–I). The tested mutations did not themselves affect DAergic neuron viability (vehicle-treated wild type and mutant nematodes had similar numbers of DAergic somas, *e.g.*, in Figure S1H) or alter the dopamine-regulated nematode food response (data not shown). These results indicate that LRK-1 protects against the DAergic neurotoxicity of 6-OHDA in nematodes.

### Human LRRK2 can functionally substitute for LRK-1 in protecting nematode DAergic neurons from 6-OHDA-induced degeneration

LRRK2 is expressed in mammalian DAergic neurons [34], [35]. Although the nematode LRRK2 homolog, LRK-1, was previously reported to have a pan-neuronal expression pattern [29], whether it is truly expressed in nematode DAergic neurons was not addressed in previous studies [8], [29]. Therefore, we generated a nematode line expressing a fusion protein composed of the N-terminus of nematode LRK-1 linked to GFP (LRK-1N::GFP, driven by the natural LRK-1 promoter) [29] and DsRed driven by the DAergic-specific promoter of *dat-1* (P*_dat-1_*), the sole dopamine transporter homolog in nematodes [33]. Analysis of these animals showed that *lrk-1* was expressed in all eight of the nematode DAergic neurons together with DsRed (Figure S2). This finding supports the possibility that LRK-1 plays a physiological role in maintenance of DAergic neuron viability.

Having shown that *lrk-1* mutant nematodes display increased sensitivity to the neurotoxic effects of 6-OHDA, we next tested whether expression of human LRRK2 could reverse this effect. We expressed WT and K1906M mutant (kinase inactive) [36] forms of human LRRK2 driven by either P*_dat-1_* or a pan-neuronal promoter (P*_H20_* [37]) in *lrk-1* mutant (km17) nematodes. Expression of WT, but not K1906M, LRRK2 in DAergic neurons was sufficient to functionally substitute for LRK-1 and rescue the cells from 6-OHDA-induced degeneration (Figure 1A). These observations indicate that LRRK2 expressed in DAergic neurons protects against neurotoxin-induced DAergic neuron degeneration, and the kinase activity of LRRK2 may be important for this role of LRRK2. Together, these data establish *C. elegans* combined with an appropriate concentration of 6-OHDA as a model to identify potential functional partners of LRRK2.

### LRK-1 signals through the PMK-1/p38 pathway to protect nematode DAergic neurons from 6-OHDA-induced degeneration

To identify the pathway through which LRRK2 acts to protect DAergic neurons from the toxicity of 6-OHDA, we tested the hypothesis that LRRK2 functions upstream of MKKs [36], [38]. We started with analysis of three major MAPK pathways: ERK, c-Jun-N-terminal kinase (JNK) and p38 [39]. Nematodes with loss-of-function mutations in the nematode homologs of ERK (*mpk-2*), JNK (*jnk-1*) and p38 (*pmk-1*) [40]–[42] did not display significant DAergic neuron degeneration in the absence of 6-OHDA (Figures 1B and S3). However, *pmk-1* mutant nematodes (Figures 1B, S3), but not *mpk-2* or *jnk-1* mutants (Figure S3 panel F), demonstrated enhanced sensitivity to 6-OHDA-induced neurotoxicity. Our finding that DAergic neurons respond to 6-OHDA similarly in *pmk-1* and *lrk-1* (*km17*) mutant nematodes suggests that p38 acts as a functional partner of LRRK2 in the maintenance of DAergic neuron viability.

Two MAPKKs lie upstream of p38, MKK3 and MKK6, and the nematode homolog of both is *sek-1* [40]. We found that nematodes with a loss-of-function mutation in *sek-1* consistently displayed enhanced susceptibility to 6-OHDA-induced neurotoxicity (Figure 1B and Figure S3 panel E). We then generated *lrk-1;pmk-1* and *lrk-1;sek-1* double mutant nematodes and found that these double mutants were no more sensitive to 6-OHDA-induced DAergic neuron degeneration than their single mutant counterparts (*lrk-1, pmk-1* or *sek-1*). They also did not exhibit a DAergic neuron degenerative phenotype in the absence of 6-OHDA (Figures 1B, S3 and data not shown). These results suggest that LRRK2, MKK3/6 and p38 function in the same genetic pathway.

Based on our finding that p38 is involved in protection of DAergic neurons from 6-OHDA-induced degeneration, we examined whether p38 becomes activated in the presence of the neurotoxin. As shown in Figure 1C, treatment of 6-OHDA for 24, but not 4 hr resulted in LRK-1-dependent phosphorylation of p38 on Thr191/Tyr193 (equivalent to Thr190/Tyr192 in humans). These data indicate that LRK-1 functions upstream of PMK-1/p38 to maintain DAergic neuron viability in the presence of 6-OHDA.

### LRRK2 acts through the p38 pathway to protect human neuroblastoma cells from the toxicity of 6-OHDA

**Figure 2:**
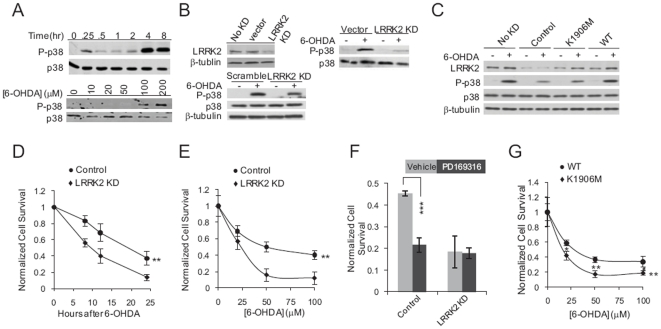
LRRK2 signaling potentiates cell survival after 6-OHDA exposure. (**A**) 6-OHDA induces two peaks of p38 phosphorylation and the later peak is 6-OHDA dose-dependent. Western blotting with antibodies against phosphorylated p38 (P-p38) and total p38. Lysates were prepared from SH-SY5Y cells treated with 100 µM 6-OHDA for the indicated times (upper panel) or with the indicated concentrations of 6-OHDA for 4 hours (lower panel). (**B**) 6-OHDA-induced p38 activation is dependent on LRRK2 function. Upper left panel: Western blot analysis of LRRK2 and β-tubulin (loading control) levels in parental SH-SY5Y cells (No KD), SH-SY5Y cells transfected with a control vector (vector), and SH-SY5Y cells from a MIX LRRK2 KD line (LRRK2 KD). Upper right panel: Western blot analysis of P-p38 and total p38 levels in SH-SY5Y cells transfected with a control vector (vector) or SH-SY5Y cells from a MIX LRRK2 KD line (LRRK2 KD) with or without exposure to 100 µM 6-OHDA for 4 hours. Lower panel: Western blot analysis of P-p38 and total p38 levels in SH-SY5Y cells transfected with a scramble shRNA (Scramble) or MIX LRRK2 shRNA (LRRK2 KD) with or without exposure to 100 µM 6-OHDA for 4 hours. (**C**) Expression of WT, but not K1906M mutant, LRRK2 restores 6-OHDA-induced p38 activation in LRRK2 KD cells. Western blot analyses of LRRK2, P-p38 and total p38 levels. Lysates were prepared from parental SH-SY5Y cells reconstituted with control vector (No KD)or 3′-UTR LRRK2 KD SH-SY5Y cells reconstituted with control vector (Control), K1906M or WT LRRK2 with or without exposure to 100 µM 6-OHDA for 4 hours. (**D**) Cells from a MIX LRRK2 KD SH-SY5Y line (LRRK2 KD, diamonds) and SH-SY5Y cells transfected with a control vector (Control, circles) were treated with 100 µM 6-OHDA for the indicated times. Cell viability was determined using an XTT-based calorimetric assay. Data represent the mean ± SEM of 3 independent experiments. **, p<0.01 by two-way ANOVA. (**E**) Cells with reduced LRRK2 expression show heightened sensitivity to 6-OHDA. Cells from a MIX LRRK2 KD SH-SY5Y line (LRRK2 KD, diamonds) and SH-SY5Y cells transfected with a control vector (Control, circles) were treated with the indicated concentrations of 6-OHDA for 24 hours. Cell viability was determined using an XTT-based calorimetric assay. Data represent the mean ± SEM of 3 (LRRK2 KD) or 4 (control) independent experiments. **, p<0.01 by two-way ANOVA. (**F**) Inhibition of p38 potentiates 6-OHDA-induced cell death in cells expressing LRRK2. SH-SY5Y cells transfected with control vector (Control) and MIX LRRK2 KD SH-SY5Y cells (LRRK2 KD) were treated with 100 µM 6-OHDA and either control vehicle or PD169316 for 24 hours. Cell survival was measured with a XTT-based calorimetric assay. Data represent the mean ± SEM of 4 independent experiments. ***, p<0.005 by t-test. (**G**) Expression of WT, but not kinase-inactive mutant, LRRK2 counteracts 6-OHDA-induced cell death. 3′-UTR LRRK2 KD SH-SY5Y cells reconstituted with K1906M mutant LRRK2 (diamonds) or WT LRRK2 (circles) were exposed to the indicated concentrations of 6-OHDA for 24 hours and cell survival was assessed with a XTT-based calorimetric assay. Data represent the mean ± SEM of 4 independent experiments. **, p<0.01 by two-way ANOVA, and *, P<0.05; **, P<0.01 by t-test.

To confirm that LRRK2 acts upstream of p38 in human cells, we chose to use the SH-SY5Y human neuroblastoma cell line since it is widely used for PD-related research and is sensitive to 6-OHDA [43]. Previously, 6-OHDA-induced p38 activation was observed in both SH-SY5Y cells and NM9D cells, another human neuroblastoma cell model of DAergic neurons [43], [44]. Here, we found that 6-OHDA induced transient activation of p38 in SH-SY5Y cells that peaked about 15 minutes after 6-OHDA exposure before disappearing and was then followed by a second stronger peak of activation at 4 hours (Figure 2A, upper panel). This second peak was 6-OHDA-dose-dependent (Figure 2A, lower panel), and was not observed in HEK293 cells (data not shown), a human kidney cell line.

To determine whether LRRK2 is required for activation of p38 following 6-OHDA exposure, we generated a number of stable SH-SY5Y-derived cell lines in which LRRK2 was knocked-down (KD) using shRNA technology. LRKK2 KD cell lines were generated by using (i) a mixture of five clones from the LRRK2 shRNA set targeting both LRRK2 coding sequences and sequences within the 3′-UTR of the LRRK2 mRNA (MIX LRRK2 KD lines) (Figures 2B and data not shown), and (ii) an shRNA targeting sequences within the LRRK2 3′-UTR only (3′-UTR LRRK2 KD lines) (Figure S5A and data not shown). The decreased LRRK2 levels in LRRK2 KD cells correlated with reduced 6-OHDA-induced p38 phosphorylation at 4 h (Figure 2B), but not at 30 min, after 6-OHDA exposure (data not shown). These data suggest that LRRK2 functions upstream of p38 after 6-OHDA exposure. The delayed interaction between LRRK2 and p38 (peaking at 4 h after drug exposure) suggests that it might be mediated by 6-OHDA-induced stress [45], [46]. We focused on this second p38 activation peak (at 4 h) for subsequent experiments.

To address whether toxins other than 6-OHDA trigger LRRK2/p38, we treated cells with H_2_O_2_, an oxidant that induces oxidative stress. In both MIX LRRK2 KD cells and SH-SY5Y cells transfected with control vector, two-phase p38 activation similar to the case of 6-OHDA exposure was detected (Figure S4). The second p38 activation was found also to be reduced in LRRK2 KD cells (Figure S4).

To examine whether the LRRK2 kinase domain is required for p38 activation following 6-OHDA treatment, we transfected cells from a 3′-UTR LRRK2 KD SH-SY5Y cell line (Figure S5A) with either WT or K1906M mutant (kinase inactive) human LRRK2. The two types of reconstituted cells had similar levels of LRRK2 (Figure 2C); however, upon 6-OHDA treatment, cells expressing WT LRRK2 showed restored p38 activation as compared to that of SH-SY5Y cells (No KD) and 3′-UTR LRRK2 KD SH-SY5Y cells transfected with a control vector. The low level of 6-OHDA-induced p38 phosphorylation observed in K1906M LRRK2-transfected LRRK2 KD cells was similar to that in unreconstituted LRRK2 KD cells (Figure 2C), suggesting that it resulted from the activity of residual endogenous LRRK2 in the LRRK2 KD cell line. Taken together, these data indicate that, as in nematodes, LRRK2 activates the p38 pathway in response to 6-OHDA exposure in human neuroblastoma cells.

### The neurotoxicity of 6-OHDA is potentiated by either knock-down of LRRK2 expression or inhibition of p38 activity

To test the impact of LRRK2 and p38 signaling on degeneration of human neurons, we examined the viability of control and MIX LRRK2 KD SH-SY5Y cells exposed to 6-OHDA with an XTT-based calorimetric assay, a method previously used to quantify 6-OHDA-induced cell death [43]. We found that 100 µM 6-OHDA induced cell death more rapidly in LRRK2 KD lines than in control cells (Figure 2D and S5E). Dose response experiments also demonstrated that LRRK2 KD cells were more sensitive to 6-OHDA than control vector-infected cells (Figure 2E). We tested the role of p38 by using a specific inhibitor of p38 kinase activity, PD169316 [47]. MIX LRRK2 KD SH-SY5Y cells and vector-transfected SH-SY5Y cells were treated with 100 µM 6-OHDA together with either control vehicle or PD169316 for 24 hours. As shown in Figure 2F, inhibition of p38 led to enhanced 6-OHDA-induced death of control SH-SY5Y cells, but not LRRK2 KD cells. It is possible that 6-OHDA already caused ∼80% death of LRRK2 KD cells, it is difficult for p38 inhibition to potentate more cell death. This observation is consistent with the concept that LRRK2 signals through p38 to protect cells against 6-OHDA toxicity. We next found that 3′-UTR LRRK2 KD SH-SY5Y cells reconstituted with K1906M mutant LRRK2 were more vulnerable to 6-OHDA-induced toxicity than 3′-UTR LRRK2 KD cells reconstituted with WT LRRK2 (Figure 2G). The smaller difference in cell survival following 6-OHDA exposure between WT-transfected and K1906M LRRK2-transfected LRRK2 KD SH-SY5Y cells as compared to the difference between control and LRRK2 KD SH-SY5Y cells (Figure 2E) is consistent with the respective differences in 6-OHDA-mediated p38 activation in the various cell lines (Figure 2B and Figure 2C). Together, these data support the concept that LRRK2 signals through p38 to protect against 6-OHDA-induced neurotoxicity and that both the signaling and function of LRRK2 and the p38 pathway are conserved between nematodes and humans.

### LRRK2 and the p38 pathway protects DAergic neurons against hαSyn-mediated degeneration

**Figure 3:**
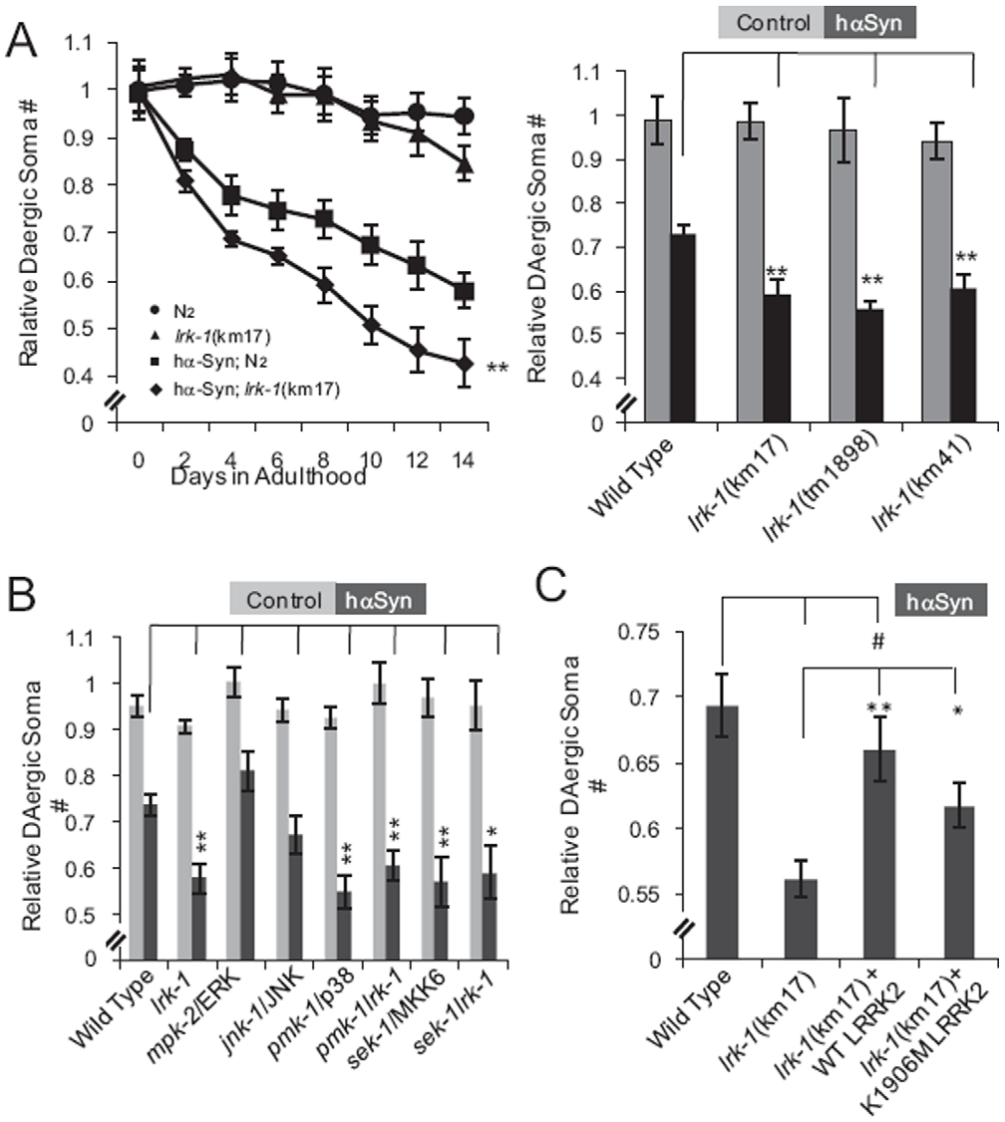
LRRK2 signaling protects against hαSyn-mediated DAergic soma degeneration in *C. elegans*. Quantification of DAergic neurons in nematodes of different genotypes with or without expression of human α-synuclein (hαSyn) in DAergic neurons. The number of DAergic somas was normalized to the mean number of DAergic somas observed in wild type (N2) L4 nematodes throughout this study, as described in Experimental Procedures. (**A**) Human α-synuclein induced death of DAergic neurons in nematodes is enhanced by mutations in components of the LRRK2 and p38 signaling pathway. Left panel, quantification of DAergic neuron degeneration as a function of age in Bristol N2 (N2, circles), loss-of-function mutant *lrk-1* (km17) (triangles), human α-synuclein (hαSyn)-expressing (in DAergic neurons) N2 (hαSyn; N2, squares), and hαSyn-expressing *lrk-1* (*km17*) (hαSyn; *lrk-1*(km17), diamonds) nematodes. The data shown represent the mean ± SEM of four independent experiments. Each experiment employed 20–30 nematodes. **, p<0.01 by two-way ANOVA. Right panel, quantification of DAergic neurons in wild type (Wild type) and different alleles of loss-of-function mutant *lrk-1* (*km17*, *tm1898* and *km41*) day 8 nematodes without (control, grey) or with (hαSyn, black) DAergic-specific expression of hαSyn. Data represent the mean ± SEM of four independent experiments. Each experiment employed 20–30 nematodes. **, p<0.01 by one-way ANOVA with Dunnett's test. For control (Wild type) and *lrk-1* (*km17*) nematodes with and without hαSyn expression, the data in the right panel corresponds to that shown in the left panel for Day 8 (the same animals produced both data sets). (**B**) *lrk-1* functions together with *pmk-1* and *sek-1* to protect against hαSyn-induced DAergic neurodegeneration. Quantification of DAergic neurons in wild type and the indicated single and double mutant day 8 nematodes without (control, grey) or with (black) DAergic-specific expression of hαSyn. Data represent the mean ± SEM of four independent experiments. Each experiment employed 20–30 nematodes. *, p<0.05; **, p<0.01; one-way ANOVA with Dunnett's test. (**C**) Human LRRK2 functionally substitutes for LRK-1 to protect nematode DAergic neurons from hαSyn-induced degeneration and LRRK2 kinase activity contributes to this protection. Quantification of DAergic neurons in day 8 nematodes including wild type (Wild type), *lrk-1* mutant (*km17*), and *km17* with pan-neuronal expression of WT (*lrk-1*+WT LRRK2) or K1906M mutant LRRK2 (*lrk-1*+K1906M LRRK2). All nematode strains also expressed hαSyn in their DAergic neurons. Data represent the mean ± SEM of four independent experiments. Each experiment employed 20–30 nematodes. ^#^, p>0.05; *, p<0.05; **, p<0.01 by one-way ANOVA with Dunnett's test.

As for neurotoxins such as 6-OHDA, hαSyn-induced death of neurons may also involve stress response pathways [48]. To further explore the physiological role of LRRK2 and the p38 signaling in neuron degeneration, we used a DAergic neuron specific hαSyn expressing nematode model that exhibits ageing-associated DAergic neuron degeneration and motor deficit, key PD pathogenic features [33]. As shown in Figures 3A–B, loss of DAergic neurons in hαSyn expressing nematodes was exacerbated in loss-of-function mutants of *lrk-1* (several different alleles), *pmk-1*/p38, and *sek-1*/MKK6 and in double mutants of *lrk-1*+*pmk-1*/p38 and *lrk-1*+*sek-1*/MKK6, but not in mutants of *mpk-2*/ERK and *jnk-1*/JNK. These data indicate that LRRK2 and the p38 signaling protects against DAergic neuron degeneration induced by both 6-OHDA and hαSyn.

Importantly, neuronal expression of human WT and kinase inactive (K1906M) LRRK2 driven by P*_H20_* in *km17* nematodes either fully or partially rescued the enhanced susceptibility of these animals to hαSyn-induced neurotoxicity (Figure 3C). This observation suggests that LRRK2 kinase activity plays a critical role in protecting DAergic neurons from hαSyn-mediated degeneration, but that a kinase-independent function of LRRK2 may also contribute to this effect. Consistent with this notion, it was previously shown that LRRK2 was required for protection of *C. elegans* [8] and *Drosophila* [21] from death induced by mitochondrial toxins, such as rotenone and paraquat, but that this effect was independent of LRRK2 kinase activity. These observations also suggest that LRRK2 mediates whole organism death induced by oxidative stress or by hαSyn DAergic neuron degeneration via distinct mechanisms, being completely or partially independent of LRRK2 kinase activity, respectively.

### LRRK2 and p38 signaling modulate GRP78 synthesis to potentiate cell survival

**Figure 4:**
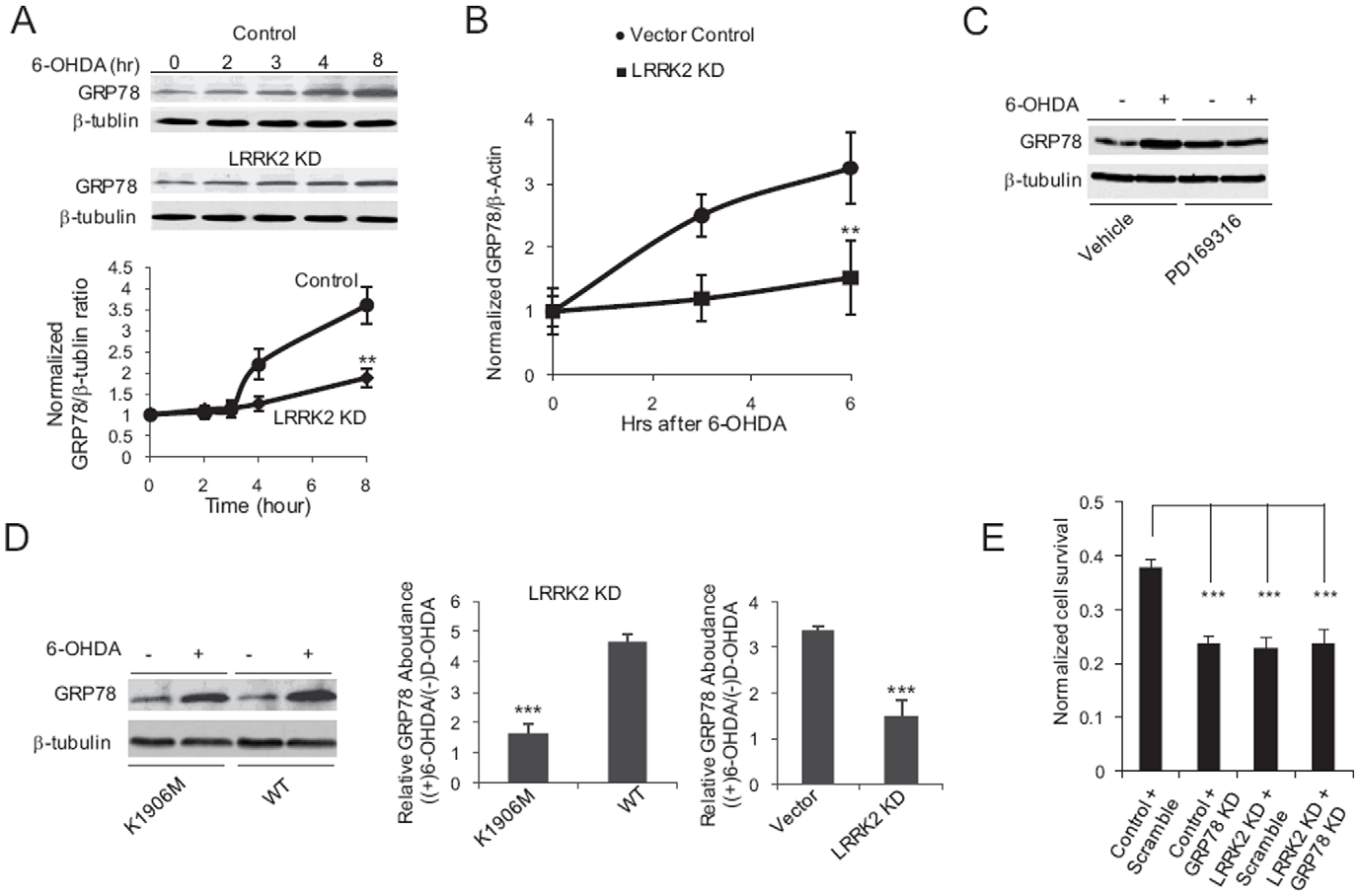
LRRK2 signaling modulates GRP78 levels to potentiate cell survival after 6-OHDA exposure. (**A**) LRRK2 is involved in 6-OHDA-mediated upregulation of GRP78 protein levels. Western blot analyses were performed on lysates from SH-SY5Y cells transfected with a control vector (Control, circles) and MIX LRRK2 KD SH-SY5Y cells (LRRK2 KD, diamonds) treated with 100 µM 6-OHDA for the indicated periods of time. Representative Western blots are shown, as well as quantization of results from 3 independent experiments. Data represent the mean ± SEM of 3 independent experiments. **, p<0.01 by two-way ANOVA. (**B**) Induction of GRP78 via 6-OHDA/LRRK2 occurs at the mRNA level. qRT-PCR quantification of GRP78 and β-actin (housekeeping control for normalization) mRNA levels was performed on SH-SY5Y cells transfected with a control vector (circles) and MIX LRRK2 KD SH-SY5Y cells (squares) treated with 100 µM 6-OHDA for the indicated periods of time. Data represent the mean ± SEM of 3 independent experiments. **: p<0.01 by two-way ANOVA. (**C**) Inhibition of p38 with PD169316 compromises 6-OHDA-induced upregulation of GRP78. Western blotting was performed on SH-SY5Y cells treated with 100 µM 6-OHDA together with 20 µM PD169316 or vehicle for 8 hours. (**D**) LRRK2 kinase activity contributes to induction of GRP78 by 6-OHDA. Western blotting was performed on 3′-UTR LRRK2 KD SH-SY5Y cells transfected with WT (WT) or K1906M mutant LRRK2 (K1906M) treated with 100 µM 6-OHDA for 8 hours. Left panel, sample blots. Middle panel, quantification of Western blot results. Right panel, under similar experimental conditions, MIX LRRK2 KD SH-SY5Y cells (LRRK2 KD) displayed compromised 6-OHDA-mediated induction of GRP78 as compared to SH-SY5Y cells transfected with control vector (vector). Data in the middle and right panels represent the mean ± SEM of 3 independent experiments. ***, p<0.005 by t-test. (**E**) shRNA-mediated double KD of GRP78 and LRRK2 displayed a similar 6-OHDA-indcuced cell death rate to that observed in single KD of either GRP78 or LRRK2. Cell survival was assessed using an XTT-based calorimetric assay in Vector control SH-SY5Y cells transfected with a scramble shRNA (Control+Scramble), or GRP78 shRNA (Control+GRP78 KD) and in MIX LRRK2 KD SH-SY5Y cells transfected with scramble shRNA (LRRK2 KD+Scramble), or GRP78 shRNA (LRRK2 KD+GRP78 KD). Cells were exposed to 100 µM 6-OHDA for 12 hours prior to assessment of cell survival. Data are shown normalized to the level of survival in cell cultures not treated with 6-OHDA and represent the mean ± SEM of 3 independent experiments. ***, p<0.005; one-way ANOVA.

Endoplasmic reticulum (ER) stress is associated with the cytotoxicity of both 6-OHDA and hαSyn in mammalian cells [46], [48]. Therefore, to further explore the biological role(s) of LRRK2 and the p38 signaling, we examined its relationship to Glucose Regulated Protein 78 (GRP78, also called Binding Immunoglobulin Protein (BiP)), an ER chaperone that is critical for cell survival in the face of ER stress [31]. We found that shRNA-mediated suppression of LRRK2 markedly compromised 6-OHDA-induced upregulation of GRP78 at both the translational (Figure 4A) and transcriptional (Figure 4B) levels. The specificity of this effect was confirmed by the finding that knock-down of LRRK2 did not affect 6-OHDA-induced transcriptional upregulation of calreticulin (Figure S6), an ER chaperone protein associated with cell death triggered by extracellular signals [49], [50]. Thus, modulation of the ER stress response by the LRRK2 signaling cascade appears to be selective. Increased levels of GRP78 were first observed ∼3 hours after addition of 6-OHDA to cell cultures, which coincided in time with LRRK2-dependent activation of p38 (Figure 2A). 6-OHDA-induced upregulation of GRP78 protein was also found to be reduced by p38 inhibition (Figure 4C) and dependent on LRRK2 kinase activity (Figure 4D). These findings suggest that GRP78 lies in the LRRK2-dependent pathway leading from 6-OHDA to cell death. Consistent with this hypothesis, shRNA-mediated knock-down of GRP78 in SH-SY5Y cells (Figure S5C) exacerbated 6-OHDA-induced cell death, and shRNA-mediated double KD of GRP78 and LRRK2 displayed a similar 6-OHDA-indcuced cell death rate to that observed in single KD of either GRP78 or LRRK2 (Figure 4E and Figure S5D). Heterologous expression of subtilase A, a functional blocker of GRP78 [51], in LRRK2 KD cells led to cell death or severe unhealthy conditions, indicating synthetic lethality (data not shown).

**Figure 5:**
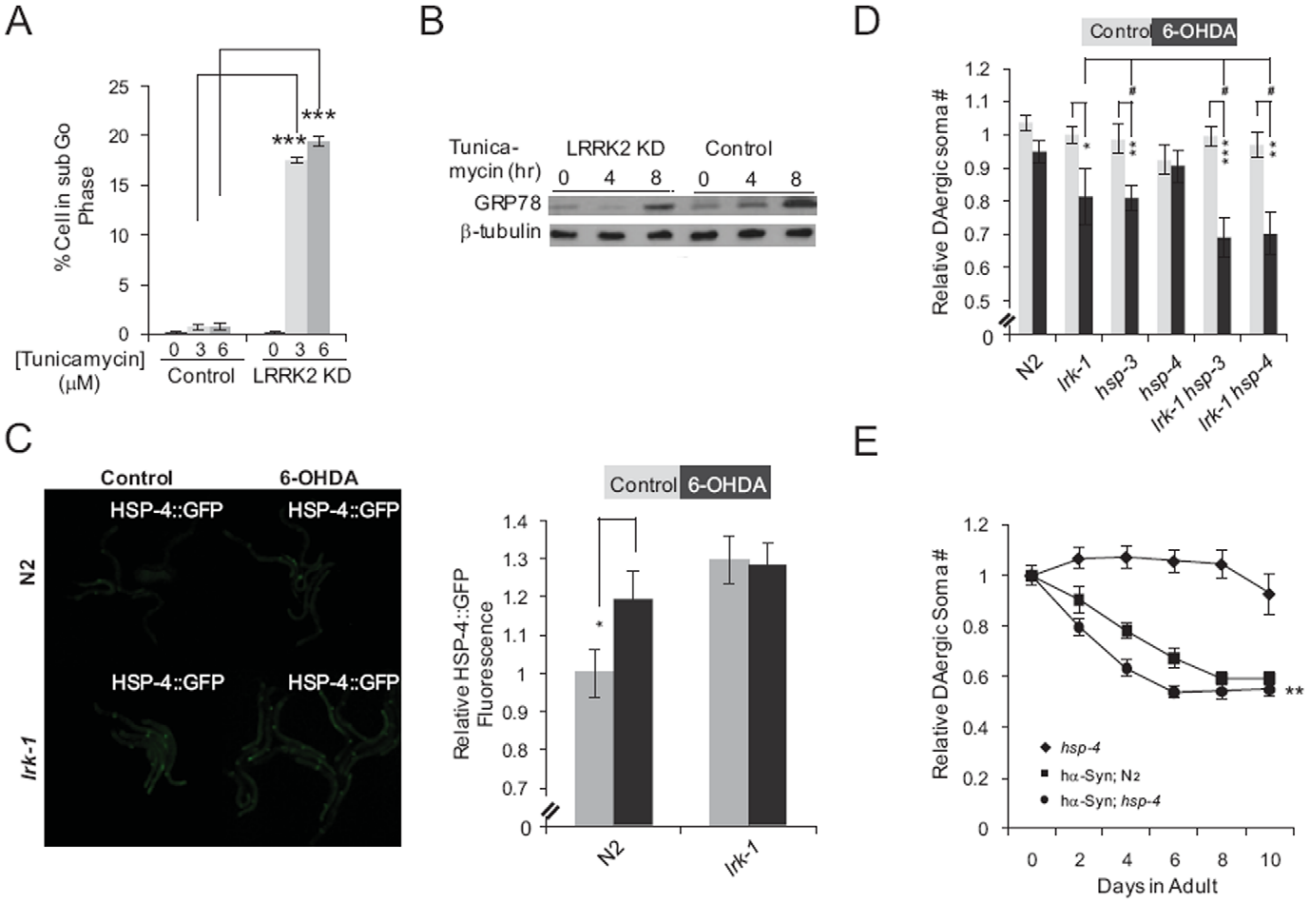
LRRK2 supports GRP78-mediated cell survival. (**A**) Quantification of the populations of cells with sub-G0 DNA content in cultures of SH-SY5Y cells transfected with a control vector (Control) and MIX LRRK2 KD SH-SY5Y cells (LRRK2 KD) treated with 0, 3 or 6 µM tunicamycin for 16 hours. Data represent the mean ± SEM of 3 independent experiments. ***, p<0.005 by t-test. (**B**) Tunicamycin-induced upregulation of GRP78 is compromised in LRRK2 KD cells. SH-SY5Y cells transfected with a control vector (Control) and MIX LRRK2 KD SH-SY5Y cells (LRRK2 KD) were treated with 3 µM tunicamycin for the indicated periods of time. Cell lysates were prepared and analyzed by Western blotting with antibodies against GRP78 and β-tubulin (loading control). (**C**) 6-OHDA induced HSP-4::GFP upregulation in N2, but not in the *lrk-1* mutant. L3 animals were treated with 2 mM 6-OHDA or vehicle alone (Control) for 1 h. Twenty-four hrs later, these animals were immobilized on 2% agarose pads with 3 mM sodium azide and then examined under a Leica DMI3000 microscope. HSP-4::GFP fluorescence of each animal was quantified as described in Experimental Procedures. Data represent the mean ± SEM. Each experiment employed 20 nematodes. *, p<0.05 by t-test. (**D**) Day 6 aniamls of the indicated genotypes were treated with vehicle alone (Control) or 2 mM 6-OHDA (6-OHDA) for 1 h at L3. DAergic neurons were counted and normalized as described in Experimental Procedures. Data represent the mean ± SEM of three independent experiments. Each experiment employed 20–30 nematodes. ^#^, p>0.05 by one-way ANOVA with Bonferroni correction, and *, p<0.05; **, p<0.01; ***, P<0.005 by t-test. (**E**) HSP-4 protects hαSyn-induced DAergic neuron degeneration in *C. elegans*. Quantification of hαSyn-induced DAergic neuron degeneration as a function of age in N2 (squares) and loss-of-function *hsp-4* mutant nematodes (circles). An *hsp-4* nematode line expressing only DsRed served as a control (diamonds). DAergic neuron somas of hermaphrodites were counted on the indicated days and normalized to the mean number of DAergic somas observed in respective L4 nematode larvae as described in Experimental Procedures. Data represent the mean ± SEM of three independent experiments. Each experiment employed 40–60 nematodes. **, p<0.01 by two-way ANOVA, testing samples between *hsp-4* and N2 nematodes expressing hαSyn.

To further investigate the link between LRRK2 signaling and ER stress, we tested the effect of tunicamycin, a widely used agent that triggers ER stress in many organisms [52], on the viability of control and MIX LRRK2 KD SH-SY5Y cells. Treatment with either 3 or 6 µM tunicamycin resulted in significant cell death in MIX LRRK2 KD, but not control SH-SY5Y cells, as evidenced by the substantial population of cells with sub-G0 DNA content in LRRK2 KD cultures exposed to this agent (Figure 5A and Figure S7A–B). Consistent with this observation, tunicamycin-induced upregulation of GRP78 was compromised in LRRK2 KD cells (Figure 5B). Based on these results, we conclude that LRRK2 signaling leads to upregulation of GRP78 synthesis, which serves to support cell survival in the face of ER stress.

Does LRRK2/LRK-1 have a functional connection with ER stress *in vivo*? We first examined the effect of 6-OHDA treatment on the abundance of HSP-4::GFP (*zcIs4*), a functional fusion protein of HSP-4, a nematode ortholog of GRP78, and GFP. It was previously demonstrated that expression of this fusion protein was induced by tunicamycin treatment or heat shock [53]. We found that 6-OHDA exposure enhanced HSP::GFP fluorescence (Figure 5C). Interestingly, fluorescence of HSP-4::GFP was significantly elevated, suggesting increased ER stress in *lrk-1* mutant nematodes, and 6-OHDA exposure did not further increase HSP::GFP fluorescence.

We next examined 6-OHDA-mediated neurodegeneration and used two loss-of-function mutants of HSP-3 and HSP-4, the only orthologs of GRP78 [51]. As shown in Figure 5D, the double mutant of *lrk-1*;*hsp-3* or *lrk-1*;*hsp-4* exhibited slightly more 6-OHDA-mediated DAergic neuron degeneration than single mutants of *lrk-1* or *hsp-3*, but not statistically significant. In addition, triple mutation of *lrk-1*;*hsp-3*;*hps-4*, but not any double mutation of these three mutations, was found to be lethal. Animals carrying this triple mutation grew slower, could not lay eggs and died before day 6, indicating synthetic lethality.

We next examined the contribution of GRP78 in hαSyn-mediated nematode DAergic neuron degeneration by testing the effect of loss-of-function mutation of HSP-4 on nematode DAergic neuron integrity maintenance, which is currently unknown. We found that hαSyn induced more severe DAergic neuron degeneration in the *hsp-4* mutant background (Figure 5E).

Taken together, we conclude that LRRK2/LRK-1 supports GRP78-mediated cell survival *in vitro* and *in vivo*.

### Expression of human pathogenic G2019S mutant LRRK2 in nematodes elicits adult-onset, ageing progressive, DAergic-specific neurodegeneration

**Figure 6:**
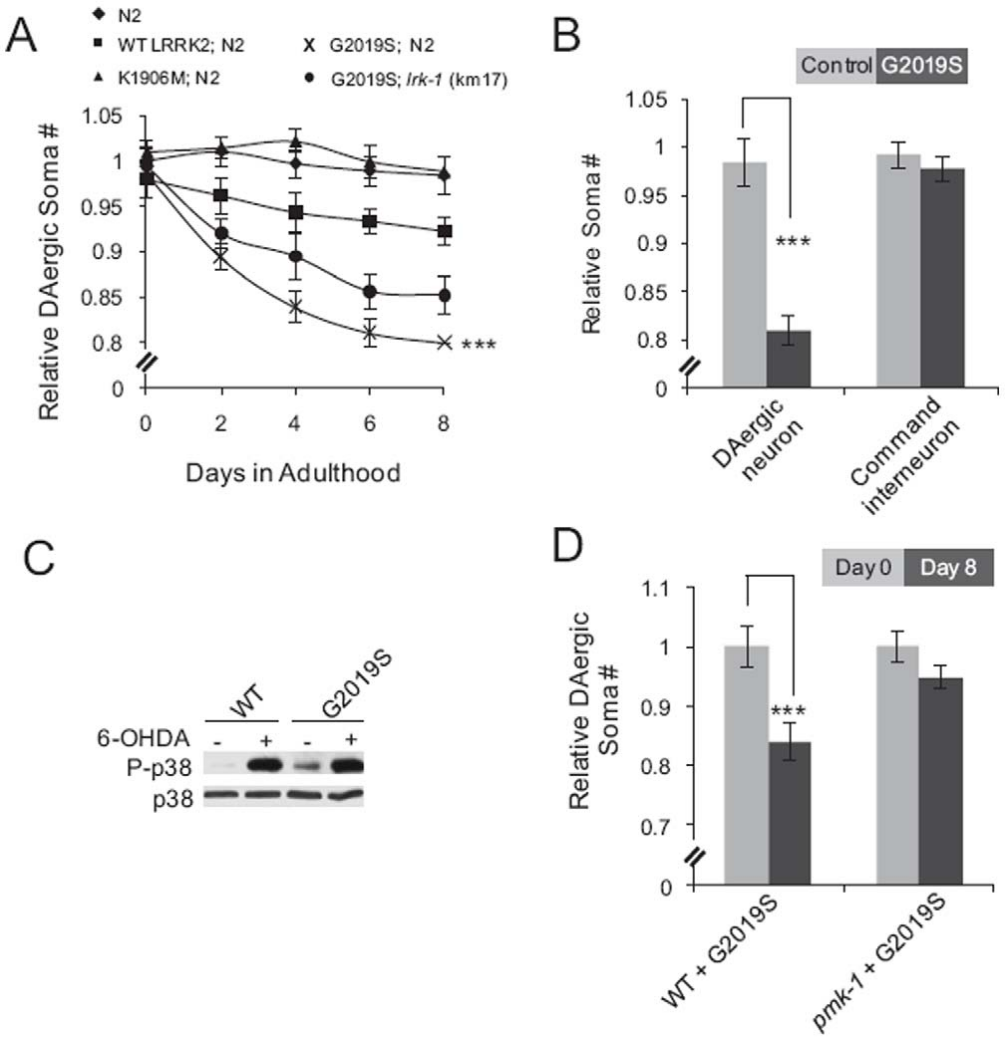
Expression of human pathogenic G2019S mutant LRRK2 in nematodes leads to ageing-associated DAergic-specific neurodegeneration and chronic p38 activation. (**A**) Quantification of DAergic neurons as a function of nematode age demonstrates that G2019S expression in nematodes elicited LRRK2 kinase-dependent DAergic neuron degeneration. Nematode variants were N2 nematodes (diamonds), N2 nematodes expressing K1906M LRRK2 (triangles), WT LRRK2 (squares), or G2019S LRRK2 (crosses) and *lrk-1* mutant nematodes (*km17*) expressing G2019S LRRK2 (circles). The number of DAergic somas was normalized to the mean number of DAergic somas observed in N2 L4 nematodes throughout the study as described in Experimental Procedures. Data represent the mean ± SEM of four independent experiments. Each experiment employed 20–30 nematodes. ***, p<0.005; two-way ANOVA compared N2 nematodes expressing G2019S mutant LRRK2 with N2 nematodes expressing WT LRRK2. (**B**) G2019S-mediated neurodegeneration in nematodes is DAergic-specific. DAergic neuron and command interneuron somas were quantified in day 8 N2 nematodes with (black) or without (grey) pan-neuronal expression of G2019S mutant LRRK2. Somas of DAergic neurons and command interneurons were visualized with P*_dat-1_* driven DsRed or P*_nmr-1_* driven YFP, respectively. Command interneurons were not DAergic. Only AVA and PVC command interneurons were used for this quantification because their fluorescent somas can be unambiguously distinguished from other command interneurons in nematodes due to their position and stronger YFP expression. The numbers of DAergic or command interneuron somas were normalized to the mean number of DAergic or command interneuron somas observed in N2 L4 nematodes, respectively (see Experimental Procedures). Data represent the mean ± SEM of four (DAergic neuron) or three (command interneuron) independent experiments. 20–30 nematodes were used for each experiment. ***, p<0.005 by t-test. (**C**) LRRK2 KD cells reconstituted with G2019S LRRK2 have a high basal level of activated p38, but are comparable to cells reconstituted with WT LRRK2 in their response to 6-OHDA. Western blotting was performed using lysates from 3′-UTR LRRK2 KD SH-SY5Y cells expressing G2019S mutant LRRK2 (G2019S) or WT LRRK2 (WT). Cells were left untreated or treated with 100 µM 6-OHDA for 4 hours. (**D**) p38 prevents G2019S-mediated adult onset DAergic neurodegeneration in nematodes. DAergic neuron somas were counted in L4 (day 0, grey) or day 8 (black) N2 or *pmk-1* mutant nematodes with pan-neuronal G2019S expression. Data was normalized to respective day 0 animals and represent the mean ± SEM of 3 independent experiments (20–30 nematodes each). ***, p<0.005 by t-test.

G2019S is the most common LRRK2 mutation associated with PD [54]. It has been shown that G2019S LRRK2 has enhanced capacity for *in vitro* phosphorylation of MKKs as compared to WT LRRK2 [36]. However, it remains unclear how this enhanced kinase activity contributes to G2019S-associated cytotoxicity. Having found that LRRK2 signaling counteracts neurodegeneration in the experiments described above, we set out to test the impact of G2019S mutant LRRK2 expression on this pathway in *C. elegans*. Therefore, we generated wild type nematode lines with pan-neuronal expression of WT LRRK2, K1906M or G2019S mutant LRRK2 (driven by the P*_H20_* promoter) as well as DAergic neuron-specific expression of DsRed and command interneuron-specific expression of yellow fluorescent protein (YFP, driven by the *nmr-1* promoter [55]). The expression of WT LRRK2, K1906M or G2019S mutant LRRK2 in wild type and *lrk-1* mutant were confirmed by qRT-PCR (Figure S8). Although G2019S LRRK2 expression in nematodes did not alter the number of identified DAergic neurons in larvae, it did result in mild but statistically significant ageing-associated progressive degeneration of DAergic neurons (Figure 6A). Command interneurons expressing G2019S did not display this phenotype (Figure 6B). In addition, pan-neuronal expression of WT or kinase inactive LRRK2 induced little or no DAergic neuron degeneration (Figure 6A). Importantly, G2019S LRRK2-mediated DAergic neuron degeneration was reduced in nematodes with loss-of-function *lrk-1* mutation (Figure 6A), although this reduction was not statistically significant. Thus, DAergic-specific neurodegeneration in G2019S LRRK2 expressing nematodes was associated with LRK-1/LRRK2 kinase activity. Although it is possible that the different expression levels of G2019S in DAergic neurons and command interneurons contributed to DAergic-specific neurodegeneration in our experiments, the obtained results are consistent with the established fact that LRRK2 is associated with PD [54].

### Chronic p38 activation contributes to G2019S-induced DAergic neurodegeration

Since the ability of LRRK2 to protect nematode DAergic neurons and human neuroblastoma cells from 6-OHDA and hαSyn was found to involve p38 activation, we examined whether p38 was also involved in promotion of DAergic neuron degeneration by G2019S mutant LRRK2. We transiently transfected 3′-UTR LRRK2 KD SH-SY5Y cells with WT or G2019S LRRK2 and found that expression of the G2019S mutant resulted in substantial basal p38 activation. The G2019S-expressing LRRK2 KD cells, but not the corresponding WT LRRK2-expressing cells, contained activated (phosphorylated) p38 without exposure to a neurotoxin (Figure 6C). Both the level of LRRK2 protein (Figure S5B) and the level of p38 activation induced by 6-OHDA (Figure 6C) was similar in the WT and G2019S reconstituted cell lines. Our finding that G2019S expression leads to constitutive p38 activation suggested that chronic p38 activation might contribute to DAergic neurodegeneration [56]. To further explore this hypothesis, we crossed a wild type nematode line expressing human G2019S LRRK2 in all neurons with a *pmk-1* knockout mutant nematode line. Animals of these two parental nematode lines exhibited similar numbers of DAergic neurons as larvae (Figure 1B and Figure 6A). Analysis of the progeny G2019S-expressing *pmk-1* mutant nematodes showed that although they had fewer identified DAergic neurons as larvae than G2019S-expressing wild type nematodes, they did not display the G2019S-mediated adult-onset DAergic neurodegeneration observed in wild type nematodes (Figure 6D). Taken together, these results indicate that chronic p38 activation contributes to pathogenic G2019S mutant LRRK2-mediated DAergic neuron degeneration in nematodes.

### Activation of p38 contributes to cytotoxicity induced by G2019S mutant LRRK2 expression in murine neurons

**Figure 7:**
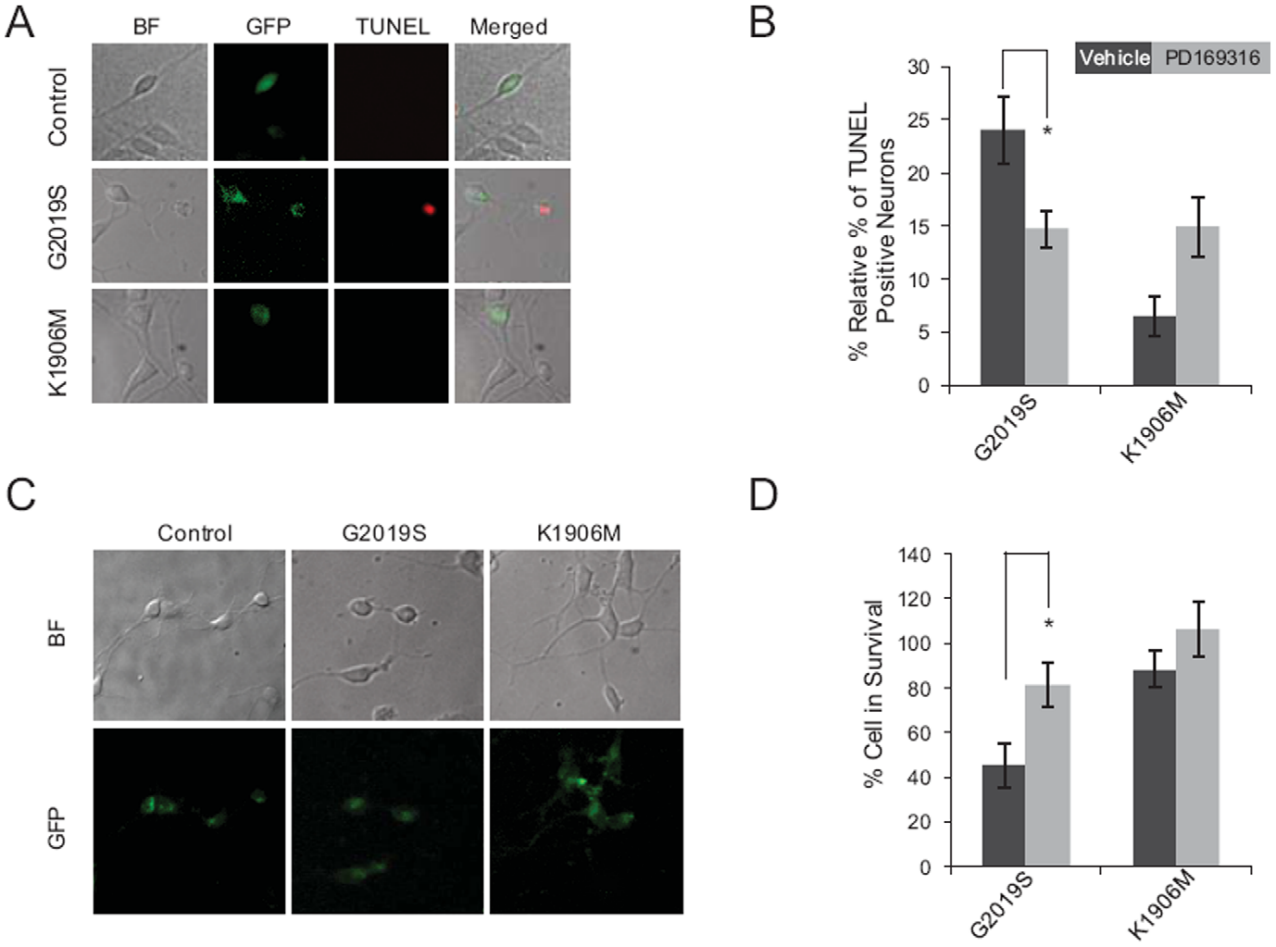
Activation of p38 contributes to cytotoxicity induced by G2019S mutant LRRK2 expression in murine neurons. Murine primary cortical neurons transfected with empty vector (control), K1906M or G2919S LRRK2 along with pEGFP vector were left untreated (vehicle) or incubated with 20 µM PD169316 for two days followed by TUNEL assay (a–b) or viability assay (C–D). (**A, C**) Representative fluorescent microscopic images of TUNEL assay (A) or viability assay (C). BF, bright field images; merged, merged images of BF, GFP and TUNEL. (**B, D**) Quantification of neuron apoptosis by TUNEL assay (B) or of neuron viability (D) demonstrates that p38 inhibition decreases the cytotoxicity of G2019S LRRK2 expressed in murine primary cortical neurons. For quantification of apoptotic cells, GFP- and TUNEL-positive neurons were counted as a percentage of total GFP-positive neurons in 40× microscopic fields. Percentages of TUNEL- positive control vector transfected neurons with or without PD169316 treatment were subtracted from the respective percentages of neurons transfected with other constructs. In viability assays, GFP-positive neurons with neurites (See Experimental Procedures) were counted as a percentage of total GFP-positive neurons. Data were normalized to neurons transfected with control vector. Data represent the mean ± SEM of three independent experiments. * p<0.05 by t-test.

To validate that activation of p38 contributes to G2019S-mediated neurodegeneration, we analyzed the effect of expression of different forms of LRRK2 on the viability of murine neurons. Cortical neurons were dissected from murine E18 embryos, transfected with expression vectors for WT, G2019S, or K1906M LRRK2 or with a control vector and then cultured for 48 h. We examined apoptosis (Figure 7A–B) and viability (Figure 7C–D) of the transfected neurons and found that expression of G2019S and WT, but not K1906M, LRRK2 markedly increased apoptosis and decreased survival of murine cortical neurons. This result is consistent with the notion that kinase activity mediates the cytotoxicity of G2019S in murine neurons [57]. In our experiments, expression of WT LRRK2 also increased apoptosis and decreased viability of cortical neurons (Figure 7A–B), presumably due to the high levels of LRRK2 kinase activity that result from overexpression of the protein. We found that p38 inhibition (using the specific chemical PD169316) potentiated death and decreased viability of neurons transfected with control vectors (data not shown), which is consistent with previous reports [58]–[60]. In contrast, p38 inhibition significantly reduced the cytotoxicity caused by G2019S or WT LRRK2 expression. Thus, exposure to PD169316 resulted in reduced apoptosis (Figure 7C) and increased viability (Figure 7D) of neurons transfected with G2019S or WT LRRK2. Based on these observations, we concluded that chronic p38 activation contributes to the cytotoxicity of G2019S LRRK2 in murine neurons.

## Discussion

Accumulated evidence in the literature indicates that multiple mechanisms may underlie LRRK2 pathology. For example, LRRK2 interacts with components involved in the autophagy-lysosomal pathway [16] or protein quality control [15], [17], modulates oxidative stress [8], [17], regulates protein synthesis [18] or mediates the microRNA pathway [19]. In this study, we identified a functional interaction between LRRK2 and GRP78, a key molecule that promotes survival of cells under ER stress. Insufficient LRRK2 kinase activity resulted in more vulnerable nematode neurons and human neuroblastoma cells, possibly through p38 signaling. Overactive LRRK2 kinase activity, however, led to nematode DAergic neuron degeneration and mammalian primary neuron death, partially through chronic p38 activation.

Specifically, we found that LRRK2 supported GRP78 upregulation in human neuroblastoma cells exposed to 6-OHDA, and that GRP78/HSP-4 protected these cells and nematode DAergic neurons against the toxicity of 6-OHDA or hαSyn. This cytoprotective mechanism of LRRK2 required its kinase activity and could execute through p38 signaling either directly or indirectly [9], [38], [61], [62], evidenced by data present in this and previous studies. For example, 6-OHDA treatment induced LRK-1-dependent p38 activity in nematodes. Moreover, *lrk-1* epistatically interacted with components of the p38 pathway in 6-OHDA- or hαSyn-mediated cell death or nematode neurodegeneration. Previously, it was also shown that p38 provides a survival signal during ER stress [52], [63]. However, an alternative explanation may exist.

While accumulated evidence has illustrated the importance of the kinase domain of LRRK2 to PD pathology, its molecular pathogenic target(s) associated with PD pathology is unknown. One possibility is that LRRK2 functions as a MAP kinase kinase kinase (MAPKKK). In support of this hypothesis, *in vitro* kinase assays demonstrated that LRRK2 catalyzes the phosphorylation of several MAP kinase kinases (MAPKK) [36]. Furthermore, LRRK2 was reported to regulate the extracellular signal-regulated kinase (ERK) pathway to attenuate H_2_O_2_-induced oxidative stress [64], [65]. In LRRK2-mediated protection against mitochondrial stress in *C. elegans*, LRRK2 interacted with MKKs [9]. Here, we found that LRRK2/LRK-1 functions upstream of p38 in maintaining DAergic neuron integrity. Contrary to the concept that LRRK2 functions as a MAPKKK, an *ex vivo* kinase substrate tracking and elucidation (KESTREL) assay identified moesin, an anchor protein between the actin cytoskeleton and the plasma membrane, as a substrate of LRRK2 [66]. A recent study showed that moesin phosphorylation by LRRK2 regulates neuronal morphogenesis by promoting actin cytoskeleton rearrangement [67]. Alternatively, LRRK2 was found to phosphorylate 4E-BP to mediate overall protein translation [12] and forkhead box transcription factor FoxO1 to enhance the transcription activity of FoxO1 [18], and directly interact with RNA-induced silencing complex (RISC) [19], all related to neurodegeneration in *Drosophila*. It is possible that LRRK2 has multiple kinase substrates, multiple functions and multiple mechanisms contributing to cell physiology.

To further define the role of LRRK2 in maintaining neuron integrity, we also overexpressed constitutive kinase active (G2019S) and kinase dead (K1906M) LRRK2 in nematodes or murine primary cortical neurons. In nematodes, we found that expression of G2019S nematodes, but not K1906, LRRK2 led to p38-dependent neurodegeneration that was reduced in a *lrk-1* mutant background. Consistently, G2019S expression in human blastoma cells activated p38 and chronic p38 activation was previously reported to be cytotoxic in cell lines [56]. It is interesting that we examined two *lrk-1* mutant nematode lines expressing G2019S, one generated from the genetic cross of a *lrk-1* mutant and a wild type nematode line expressing G2019S LRRK2; and another one generated by injecting G2019S LRRK2 construct into *lrk-1* mutant nematodes. We found that the transgenic nematode line generated by this cross displayed no DAergic neurodegeneration (Figure 6A) while the transgenic nematode line generated by injection showed some DAergic neurodegeneration (Figure 1A). The amount of G2019S LRRK2 DNA construct injected into wild type and *lrk-1* mutant nematodes to generate these two transgenic nematode lines was the same. The exact reason for this discrepancy is not clear, but it is possible that *lrk-1* mutant nematodes tolerated more injected G2019S LRRK2 construct to survive [8]. However, this more G2019S expression induced DAergic neuron degeneration. If so, this observation is consistent with the conserved function in DAergic neuron integrity maintenance between nematode LRK-1 and mammalian LRRK2. In further support of this notion, chronic p38 activation elicited by expression of G2019S LRRK2 contributed to G2019S-mediated murine primary cortical neuron death.

It is interesting that heterologous G2019S-mediated neurodegeneration was observed in DAergic neurons but not command interneurons. Although this specificity is consistent with the relevance of LRRK2 with PD, it should be noted that different levels of LRRK2 expression in these two types of neurons can also explain our results. Interestingly, a small portion of the LRK-1 N-terminus was found to promote the expression of GFP in a majority of neurons, especially emphasizing DAergic neurons [29]. It is possible that LRRK2 expression has similar behavior.

All together, our observations indicate that regulated LRRK2 activity is important for the integrity of neurons of both nematodes and mammals. Interestingly, putative kinase loss- or gain-of-function LRRK2 mutations are both associated with PD [3] and LRRK2 kinase activity has been shown to be tightly regulated [68].

It is worthy to note that neuronal expression of human WT and kinase inactive (K1906M) LRRK2 driven by P*_H20_* in *km17* nematodes rescued the enhanced sensitivity to α-synuclein neurotoxicity, fully or mildly (Figure 3D). This observation suggests that LRRK2 kinase activity plays a critical role to defend against α-synuclein-mediated DAergic neuron degeneration, but a kinase-independent role of LRRK2 may also contribute. Consistently, LRRK2-mediated protection against mitochondrial toxins, such as rotenone or paraquat, in the survival of *C. elegans* [8] or *Drosophila* [21] was found to be LRRK2 kinase dispensable. These observations also suggest that LRRK2 mediates oxidative stress-induced whole organism death and α-synuclein-mediated DAergic neuron degeneration via distinct mechanisms.

We were initially surprised to identify a functional interaction between LRRK2 and GPR78, an ER-resident chaperone that plays a central role in ER stress survival [69]. But, the cytotoxicty of 6-OHDA and hαSyn was found to associate with ER stress in mammalian cells [48] and nematodes (evidence presented here). Moreover, we found that LRRK2 also is important for human neuroblastoma cell death mediated by tunicamycin, a chemical that causes accumulation of unfolded proteins by inhibiting protein glycosylation [70]. Although the interplay between LRRK2 and GRP78 seems to be conserved between nematodes and mammals, this notion should be tested in mammalian animal models and its pathological relevance is not clear. It is worth to note that LRRK2 mainly associated with the ER of human neurons and displayed a Nissl-like pattern. Interestingly, such a Nissl-like pattern of LRRK2 was specifically disorganized in DAergic neurons of idiopathic PD cases and associated with LBs [71].

Genetic model organisms such as *C. elegans* and *Drosophila* constitute useful PD models. For example, expression of GFP in DAergic neurons led to a nematode PD model that may serve as a chemical screen platform for drugs that prevent or slow down neurotoxin-mediated DAergic neuron degeneration [25]. Heterologous expression of hαSyn-expressing PD *C. elegans* and *Drosophila* models helped understand the important pathogenic role of hαSyn [72]–[76]. Recently, heterologous expression of LRRK2 in *C. elegans* and *Drosophila* confirmed the important role of LRRK2 in mitochondrial function [8] or identified the regulation of microRNA-mediated translational repression by LRRK2 [19]. Here, we identified a functional connection between LRRK2 and GRP78 using nematode. Given their facile genetics, these *C. elegans* and *Drosophia* PD models should continuously make contributions to some aspects of PD-related research.

## Materials and Methods

### Nematode strains and maintenance

Nematodes were cultured on OP50 seeded Nematode Growth Medium (NGM) plates under standardized conditions [77]. Bristol N2 was used as the wild type strain. The following strains were obtained from either the Caenorhabditis Genetics Center (CGC): *pmk-1*(*km25*), *jnk-1*(*vc8*), *sek-1* (*km4*), *mpk-2* (*RB582*), *pek-1*(*ok275*), *hsp-3* (*ok1083*), *hsp-4* (*gk514*), and HSP-4::GFP (*zcIs4*) or the National Bioresource Project for the experimental Animal “Nematode *C. elegans*”: *lrk-1*(*tm1898*). Each strain was crossed 3 times with wild type (Bristol N2) if it had not been reported to be outcrossed by the CGC. The transgenic wild type nematode lines expressing DsRed with or without promoter of *dat-1* driven hαSyn in DAergic neurons were described previously [33], [78]. The promoters of *dat-1* or *H20* driven WT or mutant LRRK2 constructs, were injected with the co-injection marker, promoter of *nmr-1* driven YFP2 [55], into transgenic wild type nematode lines expressing DsRed with or without hαSyn (*dat-1* promoter driven) in DAergic neurons [33] to generate lines expressing LRRK2 in neurons. The promoter of *nmr-1* drives protein expression in nematode command interneurons but not in DAergic neurons.

### Nematode 6-OHDA exposure

Synchronized L3 nematodes were treated with various concentrations of 6-OHDA for 1 hour, collected and then cultured in OP50 seeded NGM plates according to a previously published protocol [25]. Neurodegenerative and biochemical experiments were performed after nematodes reached targeted ages.

### Microscopy

All confocal experiments were performed using a Leica TCS SP2 confocal microscope. The spectra used were: DsRed (λ_ex_ = 543 nm and λ_em_ = 580–630 nm) and GFP (λ_ex_ = 488 nm and λ_em_ = 510–530 nm). To count fluorescent DAergic neurons, we first immobilized living nematodes on 2% agarose pads with 3 mM sodium azide and then examined them with a Leica DMI3000 microscope according to a published method [33]. Sample images were captured with an Andor iXon^EM^ 885 EMCCD camera and SimImaging (Feng, Z. unpublished software). For HSP-4::GFP fluorescence quantification, HSP-4::GFP fluorescence of each nematode was first obtained with the freehand tool of National Instruments Vision Assistant. Background intensity was next subtracted. Twenty animals from each of the four experimental groups were examined, normalized to wild type control nematodes and graphed. All images were processed and analyzed with National Instruments Vision Assistant 8.5 (Austin, TX).

### Quantification of DAergic neurons in nematodes

DAergic neuron degeneration in nematodes was quantified by using a Leica DMI3000 microscope with 40× objective lens. A DAergic neuron was counted as surviving if its fluorescent soma was clearly observed in the expected position of the nematode body and possessed at least one dendrite that was over twice the length of its soma. We used this algorithm because DsRed puncta formed mainly in the DAergic neurites before severe DAergic soma degeneration in our hαSyn expressing nematode lines [33]. These puncta can be easily distinguished from DAergic soma by their position in the animals. A similar algorithm was previously used to quantify LRRK2-mediated primary murine neuron degeneration [57]. Because transient transgenic nematode lines were used for some of our experiments, DsRed expression in DAergic neurons could be mosaic. As a result, not all DAergic neurons displayed strong fluorescence to allow unbiased identification of DAergic somas, especially in larval stages when nematode neurons are small and their fluorescent dendrites are slim. In a small proportion of the observed nematode L4 (the 4th larvae stage), *i.e.*, day 0 nematodes, one out of the total eight DAergic somas (mostly the PDEs located in a nematode tail) in a nematode from a strain including wild type could be missed by an observer. To remove this noise, we normalized the mean DAergic soma number for 20–30 animals/strain in each independent experiment to the mean number of DAergic somas observed in similar number of wild type L4, which was obtained in the same experiment as a control. These normalizations resulted in a relative DAergic soma number over 1 for some adult nematodes of strains including Bristol N2. The relative DAergic soma number of wild type L4 nematodes in the same experiment was 1.

### Constructs

The WT human LRRK2 construct was obtained from AddGene (Cambridge, MA). The K1906M and G2019S mutant LRRK2 constructs were generated with a QuikChange® Lightning Site-Directed Mutagenesis Kit (Stratagene, Santa Clara, CA). Primers were: (sense) 5′ -gaaggagaagaagtggctgtgatgatttttaataaacatacatcac, and (antisense) 5′ –gtgatgtatgtttattaaaaatcatcacagccacttcttctccttc (K1906M); (sense) 5′-caaagattgctgactacagcattgctcagtactgc, and (antisense) 5′-gcagtactgagcaatgctgtagtcagcaatctttg (G2019S). The generated coding regions were sequenced to confirm the presence of the experimental mutations and lack of random mutations. Synthesized cDNA encoding subtilase A (YP_308822) (Genescript USA Inc., Piscataway, NJ) was subcloned into a pcDNA3.1 vector (Invitrogen) between the Kpn I and Xba I sites.

### Culture of murine embryonic cortical neurons and human neuroblastoma cells

Cortical neurons from E18 murine embryos (Swiss Webster, Charles River, Wilmington, MA) were dissociated and collected as previously described [57]. After transfection (see next section), these embryonic murine neurons were plated on laminin- (Invitrogen, Carlsbad, CA) and poly-L-lysine- (MP Biomedicals, Solon, OH) coated plates and cultured in Neurobasal Medium with addition of GlutaMAX, B-27 supplement and penicillin/streptomycin (Invitrogen). Human SH-SY5Y neuroblastoma cells were cultured in a 1∶1 mixture of modified Eagle's medium and F-12 Ham's medium with 10% fetal bovine serum and incubated in 5% CO_2_ at 37°C.

### Transfection and generation of stable shRNA LRRK2 KD cell lines

For transient LRRK2 transfections, primary murine cortical neurons or human SH-SY5Y cells were transfected using the Amaxa nucleofector system (Lonza, Switzerland) and HEK293T cells were transfected with Lipofectamine 2000 (Invitrogen) following the manufacturers' recommended protocols. Primary murine cortical neurons were cotransfected with LRRK2 expression constructs and pEGFP vector (Clontech) at a 15∶1 ratio [57]. To generate SH-SY5Y cell lines stably expressing LRRK2 or GRP78 KD, lentiviral shRNA constructs were expanded through infection of HEK293T cells cultured in 10 cm plates. Viruses were harvested and used to infect SH-SY5Y cells three times in succession. Infected SH-SY5Y cells were then cultured in the presence of puromycin (0.5 µg/ml) to select stable cell lines. The shRNAs used included: for the MIX LRRK2 KD line, a mixture of all five clones (1 µg each) from the LRRK2 shRNA set (Open Biosystems, Lafayette, CO, RHS4533-NM_198578); for the 3′ UTR LRRK2 KD line, 5 µg of clone TRCN0000021459 of RHS455-NM_198578 (mature sense sequence: CGTGTGTATGAAGGAATGTTA); for the GRP78 KD line, a mixture of all five clones (1 µg each) from the GRP78 shRNA set (Open Biosystems, RHS4533-NM_005347).

### Apoptosis and cell viability assays

Concentrated 6-OHDA (2 mM in ddH_2_O) was added to SH-SY5Y cell to achieve the indicated final concentrations. For inhibition of p38, 20 µM PD169316 (Sigma, St. Louis, MO) dissolved in DMSO or an equivalent volume of DMSO vehicle was added to wells. At the indicated times, cell survival was assessed using a Cell Proliferation Kit II (Roche, Branchburg, IN). Primary murine neurons were cultured with or without 20 µM PD169316 for 48 h after transfection. In survival assays, GFP-positive (*i.e.*, transfected) viable neurons, possessing neurites twice the length of the soma, were counted in thirty randomly selected 40× microscopic fields [57]. Apoptosis of neurons was analyzed using a TUNEL-based *in situ* cell death detection kit, TMR Red (Roche). In both apoptosis and viability assays, at least 15 neurons were counted for each microscopic field, which typically had 20–60 neurons. Numbers of TdT-mediated X-dUTP nick end labeling (TUNEL)-positive neurons relative to GFP-positive neurons were counted from ten randomly selected 40× microscopic fields. These counting analyses were performed by an investigator blind to the experimental conditions. The percentages of apoptotic or viable neurons in each experimental group relative to those in groups cotransfected with a control expression vector and pcDNA3.1-GFP (15∶1) were calculated [57].

### Flow cytometry assays

SH-SY5Y cells were cultured to 70–80% confluence and then treated for 16 hours with 3 or 6 µM tunicamycin or 400 nM thapsigargin (positive control). Cells were then washed twice with PBS, treated with 20 mg/ml RNase at 37°C for 30 min and stained with propidium iodide (50 µg/ml propidium iodide, 0.05% Triton X-100, 0.05 NaN_3_ in PBS) for 30 min at 4°C. Data were collected using a FACScan Flow Cytometer (BD Bioscience, San Jose, CA) and analyzed with WinMDI (Joe Trotter, Scripps Research Institute) and CellQuest (Becton Dickinson, Franklin Lakes, NJ) software. A small shift in the fluorescence spectrum was observed for tunicamycin treated LRRK2 KD SH-SY5Y cells, presumably due to dead cells.

### qRT-PCR

SH-SY5Y cells were plated and treated as described for cell survival assays. Total RNA from SH-SY5Y cells or nematode lines was extracted using Tri Reagent (Molecular Research Center, Cincinnati, OH) and reverse transcribed into cDNA using the High Capacity RNA to cDNA kit (Applied Biosystems, Carlsbad, CA). Primers and probes specific to the targeted genes, LRRK2 (Hs00417273_m1), calreticulin (Hs00189021_m1), GRP78 (Hs99999174_m1), β-actin (Hs99999903_m1), and Ceact-2 (GGTATGGGACAGAAGGACTCGTA-fwd, CCGTGCTCAATTGGGTACTTG-rev, and CAATCCAAGAGAGGTATCC-probe), were purchased from Applied Biosystems, Calsbad, CA, and used to perform real-time quantitative RT-PCR on a StepOne Plus instrument (Applied Biosystems) under standard conditions. The ΔΔCt for each sample was determined using StepOne Software v2.1 (Applied Biosystems).

### Western blots

For Western blotting of nematode samples, animals were washed three times with M9 buffer [77] and lysed in HB buffer containing a protease inhibitor and phosphatase inhibitor mixture (Roche) followed by brief sonication [40]. For human cells, cells were treated in 10 cm plates and then collected as described for cell survival assays. Cells then were washed with PBS, harvested and lysed in ratio immunoprecipitation assay (RIPA) buffer containing a protease inhibitor and phosphatase inhibitor mixture. Insoluble cell debris was removed by centrifugation (13,000 rpm, 10 min) with a benchtop centrifuge at 4°C and the resulting supernatants were used for Western blotting. The antibodies used were: anti-nematode p38 (self-developed by N.H. and K.M.) [40]; anti-p38, phospho-specific anti-P-p38, anti-GRP78, anti-eIF2α, phospho-specific anti-P-eIF2α, and anti-MKK6 (Cell Signaling, Danvers, MA); and anti-LRRK2 (Sigma). Antibodies against P-p38 from Cell Signaling were demonstrated previously to specifically recognize nematode P-p38, but not un-phosphorylated p38 [40]. Quantification of Western blot results were obtained using National Instruments Vision Assistant 8.5.

### Statistical analysis

Statistical significance was analyzed using Statistica software (StatSoft, Tulsa, OK). T-tests, ANOVA with Bonferroni corrections or Dunnet's post-hoc analyses were used for their appropriate applications as indicated in the Figure legends.

## Supporting Information

Figure S1 **6-OHDA-induced DAergic soma degeneration in** ***C.elegans*** **.** (**A–F**) 6-OHDA induces degeneration of nematode DAergic neurons. Day 6 wild type nematodes with DsRed-marked DAergic neurons were left untreated (control) or treated with 1 mM 6-OHDA alone or in combination with 1 mM imipramine. Details are described in Experimental procedures. Representative DsRed fluorescent (top panels) and bright field (D–F) images are shown with DAergic neurons located in the nematode head (CEPs and ADEs) indicated by arrows. (**G–I**) Quantification of 6-OHDA-induced DAergic neuron degeneration as a function of age in wild type and loss-of-function *lrk-1* mutant (*km17*) nematodes. Wild type (g, i) and *lrk-1* mutant (*km17*) L3 nematodes (h, i) were incubated in solutions containing either vehicle (control, red), 2 mM 6-OHDA (black) or 2 mM 6-OHDA+1 mM imipramine (green) for 1 hour. DAergic neuron somas of hermaphrodites were counted on the indicated days and normalized to the mean number of DAergic somas observed in wild type L4 nematode larvae (See details in Experimental Procedures). Data represent the mean ± SEM of four independent experiments. Each experiment employed 20–30 nematodes. **, p<0.01 and ***, p<0.005 by two-way ANOVA.
(TIF)

Figure S2 **LRK-1 is expressed in nematode DAergic neurons.** (**a–c**) Representative images of LRK-1N::GFP fusion protein (a), DsRed marking DAergic neurons (b), and merged images of a and b (c) demonstrate that LRK-1 is expressed in all three types of nematode DAergic neurons: CEPs (cephalic, upper panels), ADEs (anterior deirid, middle panels) and PDEs (posterior deirid, lower panels). Images show day 1 wild type nematode expressing LRK-1N::GFP and P*_dat-1_* driven DsRed. Living nematodes were immobilized with azide on agarose pads, and images were taken with a Leica cofocal microscope (See Experimental Procedures).
(TIF)

Figure S3 **LRK-1 signals through p38 MAP kinase to protect DAergic neurons against 6-OHDA-induced neurotoxicity.** (**A–E**) DAergic neuron degeneration is shown as a function of age in wild type (a), *lrk-1*/LRRK2 loss-of-function mutant (*km17*) (b), *pmk-1*/p38 *null* mutant (c), *lrk-1*(km17)*pmk-1(null)* double mutant (d) and *sek-1*/MKK6 *null* (e) nematodes incubated in solutions containing either control vehicle (red), 2 mM 6-OHDA (black) or 2 mM 6-OHDA+1 mM imipramine (green). Experimental details are provided in the Experimental Procedures section. Data represent means ± SEM of three independent experiments. Each experiment employed 20–30 nematodes. (**F**) Quantification of DAergic neuron degeneration in wild type, *lrk-1* loss-of-function, *jnk-1*/JNK *null*, and *mek-2*/ERK *null* day 4 nematodes exposed to 2 mM 6-OHDA or control vehicle. Experimental details are provided in the Experimental Procedures section. Data represent means ± SEM of three independent experiments. Nematode numbers (n) varied from 10–30 in each experiment. ***, p<0.005; t-test.
(TIF)

Figure S4 **LRRK2 KD cells show similar activation of p38 under H_2_O_2_ exposure.** Both MIX LRRK2 KD cells (LRRK2 KD) and SH-SY5Y vector control (control) cells were treated with 20 µM H_2_O_2_ for the indicated time. Western blot shows similar activation of p38 in both cell lines.
(TIF)

Figure S5 **shRNA-mediated knock-down (KD) of LRRK2 and GRP78 expression in human SH-SY5Y cells.** (**A**) Western blots show that 3′-UTR LRRK2 KD SH-SY5Y cells (LRRK2 KD) exhibited low levels of LRRK2 compared to SH-SY5Y cells without LRRK2 KD (No KD) or SH-SY5Y cells transfected with control vector (vector). (**B**) Western blots show that 3′-UTR LRRK2 KD SH-SY5Y cells transfected with WT (LRRK2 KD+WT), K1906M mutant (LRRK2 KD+K1906M) and G2019S mutant (LRRK2 KD+G2019S) LRRK2 evidenced similar levels of LRRK2. (**C**) Western blots show that SH-SY5Y cells transfected with a GRP78 shRNA set (GRP78 KD) exhibited less GRP78 expression than SH-SY5Y cells transfected with control vector (Vector). (**D**) Cells lacking GRP78 expression show increased sensitivity to 6-OHDA. Cell survival was assessed using an XTT-based calorimetric assay in SH-SY5Y cells transfected with a control vector (control, circles) and in MIX GRP78 KD SH-SY5Y cells (GRP78 KD, squares). Cells were exposed to the indicated concentrations of 6-OHDA for 24 hours prior to assessment of cell survival. Data are shown normalized to the level of survival in cell cultures not treated with 6-OHDA and represent the mean ± SEM of 3 independent experiments. *, p<0.05; two-way ANOVA. (**E**) Cells from a MIX LRRK2 KD SH-SY5Y line (LRRK2 KD) and SH-SY5Y cells transfected with a scramble shRNA (Scramble) were treated with 100 µM 6-OHDA for 12 hours. Cell viability was determined using an XTT-based calorimetric assay. Data represent the mean ± SEM of 3 independent experiments. ***, p<0.005 by t-test.
(TIF)

Figure S6 **LRRK2 expression does not affect 6-OHDA-mediated induction of calreticulin transcription in SH-SY5Y cells.** qPCR quantification of calreticulin induced by exposure to 100 µM 6-OHDA was compared between SH-SY5Y cells transfected with control vector (circles) and MIX LRRK2 KD (squares). X-axis shows periods of 6-OHDA exposure. Cells were exposed to 6-OHDA for specified periods and total RNA was extracted and transcribed into cDNA, followed by qPCR experiments. Details are provided in the Experimental Procedures section. Data represent the means ± SEM of 3 independent experiments. ^#^, p>0.05 by two-way ANOVA.
(TIF)

Figure S7 **LRRK2 supports GRP78-mediated cell survival against tunicamycin.** (**A**) LRRK2 KD cells are more vulnerable to tunicamycin-induced cell death than wild type cells. Sample flow cytometric traces of propidium iodide-stained control SH-SY5Y cells (Control) (A) or LRRK2 MIX KD SH-SY5Y cells (LRRK2 KD) (**B**) with (right panel) or without (left panel) 3 µM tunicamycin exposure for 16 hours. M1, M2 and M3 indicate cells with DNA content corresponding to the G0/G1 phase of the cell cycle, sub-G0 (apoptotic), and all other phases, respectively. FL2-H indicates the channel used to observe fluorescence.
(TIF)

Figure S8 **LRRK2 qRT-PCR confirmed the expression of WT LRRK2 and mutant forms K1906M and G2019S in wild type and** ***lrk-1*** **mutant backgrounds.** Wild type nematode lines and *lrk-1* mutant nematode lines with pan-neuronal expression of WT LRRK2, K1906M or G2019S mutant LRRK2 (driven by the P*_H20_* promoter) as well as DAergic neuron-specific expression of DsRed and command interneuron-specific expression of yellow fluorescent protein (YFP, driven by the *nmr-1* promoter) were subjected to total RNA extraction, and qRT-PCR analysis. The relative quantity (RQ) value of WT LRRK2, K1906M or G2019S mutant LRRK2 vs. actin-1 were calculated. The error bar was based on the RQ_Min/Max_ confidence level that represents the standard error of the mean expression level (RQ value).
(TIF)

## References

[1] Zimprich A, Biskup S, Leitner P, Lichtner P, Farrer M (2004). Mutations in LRRK2 cause autosomal-dominant parkinsonism with pleomorphic pathology.. Neuron.

[2] Paisan-Ruiz C, Jain S, Evans EW, Gilks WP, Simon J (2004). Cloning of the gene containing mutations that cause PARK8-linked Parkinson's disease.. Neuron.

[3] Paisan-Ruiz C (2009). LRRK2 gene variation and its contribution to Parkinson disease.. Hum Mutat.

[4] West AB, Moore DJ, Biskup S, Bugayenko A, Smith WW (2005). Parkinson's disease-associated mutations in leucine-rich repeat kinase 2 augment kinase activity.. Proc Natl Acad Sci U S A.

[5] Lee BD, Shin JH, Vankampen J, Petrucelli L, West AB (2010). Inhibitors of leucine-rich repeat kinase-2 protect against models of Parkinson's disease.. Nat Med.

[6] Dauer W, Ho CC (2010). The biology and pathology of the familial Parkinson's disease protein LRRK2.. Mov Disord.

[7] Greggio E, Cookson MR (2009). Leucine-rich repeat kinase 2 mutations and Parkinson's disease: three questions.. ASN Neuro.

[8] Saha S, Guillily MD, Ferree A, Lanceta J, Chan D (2009). LRRK2 modulates vulnerability to mitochondrial dysfunction in Caenorhabditis elegans.. J Neurosci.

[9] Hsu CH, Chan D, Greggio E, Saha S, Guillily MD (2010). MKK6 binds and regulates expression of Parkinson's disease-related protein LRRK2.. J Neurochem.

[10] Liu Z, Wang X, Yu Y, Li X, Wang T (2008). A Drosophila model for LRRK2-linked parkinsonism.. Proc Natl Acad Sci U S A.

[11] Tain LS, Mortiboys H, Tao RN, Ziviani E, Bandmann O (2009). Rapamycin activation of 4E-BP prevents parkinsonian dopaminergic neuron loss.. Nat Neurosci.

[12] Imai Y, Gehrke S, Wang HQ, Takahashi R, Hasegawa K (2008). Phosphorylation of 4E-BP by LRRK2 affects the maintenance of dopaminergic neurons in Drosophila.. EMBO J.

[13] Tong Y, Pisani A, Martella G, Karouani M, Yamaguchi H (2009). R1441C mutation in LRRK2 impairs dopaminergic neurotransmission in mice.. Proc Natl Acad Sci U S A.

[14] Li Y, Liu W, Oo TF, Wang L, Tang Y (2009). Mutant LRRK2(R1441G) BAC transgenic mice recapitulate cardinal features of Parkinson's disease.. Nat Neurosci.

[15] Ko HS, Bailey R, Smith WW, Liu Z, Shin JH (2009). CHIP regulates leucine-rich repeat kinase-2 ubiquitination, degradation, and toxicity.. Proc Natl Acad Sci U S A.

[16] Tong Y, Yamaguchi H, Giaime E, Boyle S, Kopan R (2010). Loss of leucine-rich repeat kinase 2 causes impairment of protein degradation pathways, accumulation of alpha-synuclein, and apoptotic cell death in aged mice.. Proc Natl Acad Sci U S A.

[17] Ng CH, Mok SZ, Koh C, Ouyang X, Fivaz ML (2009). Parkin protects against LRRK2 G2019S mutant-induced dopaminergic neurodegeneration in Drosophila.. J Neurosci.

[18] Kanao T, Venderova K, Park DS, Unterman T, Lu B (2010). Activation of FoxO by LRRK2 induces expression of proapoptotic proteins and alters survival of postmitotic dopaminergic neuron in Drosophila.. Hum Mol Genet.

[19] Gehrke S, Imai Y, Sokol N, Lu B (2010). Pathogenic LRRK2 negatively regulates microRNA-mediated translational repression.. Nature.

[20] Lee SB, Kim W, Lee S, Chung J (2007). Loss of LRRK2/PARK8 induces degeneration of dopaminergic neurons in Drosophila.. Biochem Biophys Res Commun.

[21] Wang D, Tang B, Zhao G, Pan Q, Xia K (2008). Dispensable role of Drosophila ortholog of LRRK2 kinase activity in survival of dopaminergic neurons.. Mol Neurodegener.

[22] Li X, Patel JC, Wang J, Avshalumov MV, Nicholson C (2010). Enhanced striatal dopamine transmission and motor performance with LRRK2 overexpression in mice is eliminated by familial Parkinson's disease mutation G2019S.. J Neurosci.

[23] Spillantini MG, Schmidt ML, Lee VM, Trojanowski JQ, Jakes R (1997). Alpha-synuclein in Lewy bodies.. Nature.

[24] Polymeropoulos MH, Lavedan C, Leroy E, Ide SE, Dehejia A (1997). Mutation in the alpha-synuclein gene identified in families with Parkinson's disease.. Science.

[25] Nass R, Hall DH, Miller DM, Blakely RD (2002). Neurotoxin-induced degeneration of dopamine neurons in Caenorhabditis elegans.. Proc Natl Acad Sci U S A.

[26] Ungerstedt U (1968). 6-Hydroxy-dopamine induced degeneration of central monoamine neurons.. Eur J Pharmacol.

[27] Burns RS, Chiueh CC, Markey SP, Ebert MH, Jacobowitz DM (1983). A primate model of parkinsonism: selective destruction of dopaminergic neurons in the pars compacta of the substantia nigra by N-methyl-4-phenyl-1,2,3,6-tetrahydropyridine.. Proc Natl Acad Sci U S A.

[28] Lin X, Parisiadou L, Gu XL, Wang L, Shim H (2009). Leucine-rich repeat kinase 2 regulates the progression of neuropathology induced by Parkinson's-disease-related mutant alpha-synuclein.. Neuron.

[29] Sakaguchi-Nakashima A, Meir JY, Jin Y, Matsumoto K, Hisamoto N (2007). LRK-1, a C. elegans PARK8-related kinase, regulates axonal-dendritic polarity of SV proteins.. Curr Biol.

[30] Yao C, El Khoury R, Wang W, Byrd TA, Pehek EA (2010). LRRK2-mediated neurodegeneration and dysfunction of dopaminergic neurons in a Caenorhabditis elegans model of Parkinson's disease.. Neurobiol Dis.

[31] Harding HP, Zhang Y, Ron D (1999). Protein translation and folding are coupled by an endoplasmic-reticulum-resident kinase.. Nature.

[32] Lundblad M, Andersson M, Winkler C, Kirik D, Wierup N (2002). Pharmacological validation of behavioural measures of akinesia and dyskinesia in a rat model of Parkinson's disease.. Eur J Neurosci.

[33] Cao PX, Yuan YY, Pehek EA, R. MA, Huang Y (2010). Alpha-Synuclein Disrupted Dopamine Homeostasis Leads to Dopaminergic Neuron Degeneration in *Caenorhabditis elegans*.. PLoS ONE.

[34] Melrose H, Lincoln S, Tyndall G, Dickson D, Farrer M (2006). Anatomical localization of leucine-rich repeat kinase 2 in mouse brain.. Neuroscience.

[35] Simon-Sanchez J, Herranz-Perez V, Olucha-Bordonau F, Perez-Tur J (2006). LRRK2 is expressed in areas affected by Parkinson's disease in the adult mouse brain.. Eur J Neurosci.

[36] Gloeckner CJ, Schumacher A, Boldt K, Ueffing M (2009). The Parkinson disease-associated protein kinase LRRK2 exhibits MAPKKK activity and phosphorylates MKK3/6 and MKK4/7, in vitro.. J Neurochem.

[37] Yabe T, Suzuki N, Furukawa T, Ishihara T, Katsura I (2005). Multidrug resistance-associated protein MRP-1 regulates dauer diapause by its export activity in Caenorhabditis elegans.. Development.

[38] Hsu CH, Chan D, Wolozin B (2010). LRRK2 and the Stress Response: Interaction with MKKs and JNK-Interacting Proteins.. Neurodegener Dis.

[39] Krishna M, Narang H (2008). The complexity of mitogen-activated protein kinases (MAPKs) made simple.. Cell Mol Life Sci.

[40] Kim DH, Feinbaum R, Alloing G, Emerson FE, Garsin DA (2002). A conserved p38 MAP kinase pathway in Caenorhabditis elegans innate immunity.. Science.

[41] Kawasaki M, Hisamoto N, Iino Y, Yamamoto M, Ninomiya-Tsuji J (1999). A Caenorhabditis elegans JNK signal transduction pathway regulates coordinated movement via type-D GABAergic motor neurons.. EMBO J.

[42] Lackner MR, Kornfeld K, Miller LM, Horvitz HR, Kim SK (1994). A MAP kinase homolog, mpk-1, is involved in ras-mediated induction of vulval cell fates in Caenorhabditis elegans.. Genes Dev.

[43] Choi WS, Eom DS, Han BS, Kim WK, Han BH (2004). Phosphorylation of p38 MAPK induced by oxidative stress is linked to activation of both caspase-8- and -9-mediated apoptotic pathways in dopaminergic neurons.. J Biol Chem.

[44] Gomez-Lazaro M, Galindo MF, Concannon CG, Segura MF, Fernandez-Gomez FJ (2008). 6-Hydroxydopamine activates the mitochondrial apoptosis pathway through p38 MAPK-mediated, p53-independent activation of Bax and PUMA.. J Neurochem.

[45] Yamamuro A, Yoshioka Y, Ogita K, Maeda S (2006). Involvement of endoplasmic reticulum stress on the cell death induced by 6-hydroxydopamine in human neuroblastoma SH-SY5Y cells.. Neurochem Res.

[46] Ryu EJ, Harding HP, Angelastro JM, Vitolo OV, Ron D (2002). Endoplasmic reticulum stress and the unfolded protein response in cellular models of Parkinson's disease.. J Neurosci.

[47] Kummer JL, Rao PK, Heidenreich KA (1997). Apoptosis induced by withdrawal of trophic factors is mediated by p38 mitogen-activated protein kinase.. J Biol Chem.

[48] Smith WW, Jiang H, Pei Z, Tanaka Y, Morita H (2005). Endoplasmic reticulum stress and mitochondrial cell death pathways mediate A53T mutant alpha-synuclein-induced toxicity.. Hum Mol Genet.

[49] Holtz WA, O'Malley KL (2003). Parkinsonian mimetics induce aspects of unfolded protein response in death of dopaminergic neurons.. J Biol Chem.

[50] Lee YM, Park SH, Chung KC, Oh YJ (2003). Proteomic analysis reveals upregulation of calreticulin in murine dopaminergic neuronal cells after treatment with 6-hydroxydopamine.. Neurosci Lett.

[51] Nakajima S, Hiramatsu N, Hayakawa K, Saito Y, Kato H (2011). Selective Abrogation of BiP/GRP78 Blunts Activation of NF-{kappa}B through the ATF6 Branch of the UPR: Involvement of C/EBP{beta} and mTOR-Dependent Dephosphorylation of Akt.. Mol Cell Biol.

[52] Bischof LJ, Kao CY, Los FC, Gonzalez MR, Shen Z (2008). Activation of the unfolded protein response is required for defenses against bacterial pore-forming toxin in vivo.. PLoS Pathog.

[53] Calfon M, Zeng H, Urano F, Till JH, Hubbard SR (2002). IRE1 couples endoplasmic reticulum load to secretory capacity by processing the XBP-1 mRNA.. Nature.

[54] Kachergus J, Mata IF, Hulihan M, Taylor JP, Lincoln S (2005). Identification of a novel LRRK2 mutation linked to autosomal dominant parkinsonism: evidence of a common founder across European populations.. Am J Hum Genet.

[55] Brockie PJ, Mellem JE, Hills T, Madsen DM, Maricq AV (2001). The C. elegans glutamate receptor subunit NMR-1 is required for slow NMDA-activated currents that regulate reversal frequency during locomotion.. Neuron.

[56] Kong AN, Yu R, Chen C, Mandlekar S, Primiano T (2000). Signal transduction events elicited by natural products: role of MAPK and caspase pathways in homeostatic response and induction of apoptosis.. Arch Pharm Res.

[57] Smith WW, Pei Z, Jiang H, Dawson VL, Dawson TM (2006). Kinase activity of mutant LRRK2 mediates neuronal toxicity.. Nat Neurosci.

[58] Krysov SV, Rowley TF, Al-Shamkhani A (2007). Inhibition of p38 mitogen-activated protein kinase unmasks a CD30-triggered apoptotic pathway in anaplastic large cell lymphoma cells.. Mol Cancer Ther.

[59] Varghese J, Chattopadhaya S, Sarin A (2001). Inhibition of p38 kinase reveals a TNF-alpha-mediated, caspase-dependent, apoptotic death pathway in a human myelomonocyte cell line.. J Immunol.

[60] Tourian L, Zhao H, Srikant CB (2004). p38alpha, but not p38beta, inhibits the phosphorylation and presence of c-FLIPS in DISC to potentiate Fas-mediated caspase-8 activation and type I apoptotic signaling.. J Cell Sci.

[61] Kumar A, Greggio E, Beilina A, Kaganovich A, Chan D (2010). The Parkinson's disease associated LRRK2 exhibits weaker in vitro phosphorylation of 4E-BP compared to autophosphorylation.. PLoS One.

[62] White LR, Toft M, Kvam SN, Farrer MJ, Aasly JO (2007). MAPK-pathway activity, Lrrk2 G2019S, and Parkinson's disease.. J Neurosci Res.

[63] Ranganathan AC, Zhang L, Adam AP, Aguirre-Ghiso JA (2006). Functional coupling of p38-induced up-regulation of BiP and activation of RNA-dependent protein kinase-like endoplasmic reticulum kinase to drug resistance of dormant carcinoma cells.. Cancer Res.

[64] Heo HY, Park JM, Kim CH, Han BS, Kim KS (2009). LRRK2 enhances oxidative stress-induced neurotoxicity via its kinase activity.. Exp Cell Res.

[65] Liou AK, Leak RK, Li L, Zigmond MJ (2008). Wild-type LRRK2 but not its mutant attenuates stress-induced cell death via ERK pathway.. Neurobiol Dis.

[66] Jaleel M, Nichols RJ, Deak M, Campbell DG, Gillardon F (2007). LRRK2 phosphorylates moesin at threonine-558: characterization of how Parkinson's disease mutants affect kinase activity.. Biochem J.

[67] Parisiadou L, Xie C, Cho HJ, Lin X, Gu XL (2009). Phosphorylation of ezrin/radixin/moesin proteins by LRRK2 promotes the rearrangement of actin cytoskeleton in neuronal morphogenesis.. J Neurosci.

[68] Greggio E, Taymans JM, Zhen EY, Ryder J, Vancraenenbroeck R (2009). The Parkinson's disease kinase LRRK2 autophosphorylates its GTPase domain at multiple sites.. Biochem Biophys Res Commun.

[69] Ma K, Vattem KM, Wek RC (2002). Dimerization and release of molecular chaperone inhibition facilitate activation of eukaryotic initiation factor-2 kinase in response to endoplasmic reticulum stress.. J Biol Chem.

[70] Leavitt R, Schlesinger S, Kornfeld S (1977). Tunicamycin inhibits glycosylation and multiplication of Sindbis and vesicular stomatitis viruses.. J Virol.

[71] Vitte J, Traver S, Maues De Paula A, Lesage S, Rovelli G (2010). Leucine-rich repeat kinase 2 is associated with the endoplasmic reticulum in dopaminergic neurons and accumulates in the core of Lewy bodies in Parkinson disease.. J Neuropathol Exp Neurol.

[72] Kuwahara T, Koyama A, Gengyo-Ando K, Masuda M, Kowa H (2006). Familial Parkinson mutant alpha-synuclein causes dopamine neuron dysfunction in transgenic Caenorhabditis elegans.. J Biol Chem.

[73] Chen L, Feany MB (2005). Alpha-synuclein phosphorylation controls neurotoxicity and inclusion formation in a Drosophila model of Parkinson disease.. Nat Neurosci.

[74] Periquet M, Fulga T, Myllykangas L, Schlossmacher MG, Feany MB (2007). Aggregated alpha-synuclein mediates dopaminergic neurotoxicity in vivo.. J Neurosci.

[75] Outeiro TF, Kontopoulos E, Altmann SM, Kufareva I, Strathearn KE (2007). Sirtuin 2 inhibitors rescue alpha-synuclein-mediated toxicity in models of Parkinson's disease.. Science.

[76] Cooper AA, Gitler AD, Cashikar A, Haynes CM, Hill KJ (2006). Alpha-synuclein blocks ER-Golgi traffic and Rab1 rescues neuron loss in Parkinson's models.. Science.

[77] Brenner S (1974). The genetics of Caenorhabditis elegans.. Genetics.

[78] Wragg RT, Hapiak V, Miller SB, Harris GP, Gray J (2007). Tyramine and octopamine independently inhibit serotonin-stimulated aversive behaviors in Caenorhabditis elegans through two novel amine receptors.. J Neurosci.

