# Supplementary figures and images for: Dysregulated LRRK2 Signaling in Response to Endoplasmic Reticulum Stress Leads to Dopaminergic Neuron Degeneration in C. elegans
Source: PLoS One. 2011 Aug 3;6(8):e22354. doi: 10.1371/journal.pone.0022354 (PMC3153934; doi:10.1371/journal.pone.0022354)

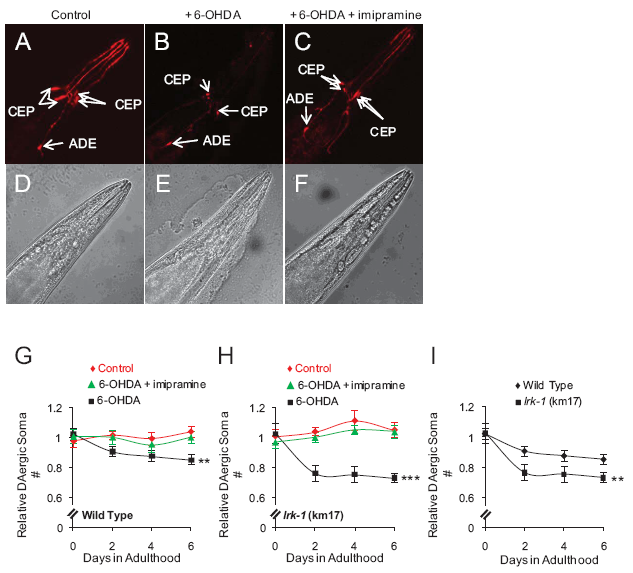

Supplement: Figure S1 — 6-OHDA-induced DAergic soma degeneration in C.elegans . (A–F) 6-OHDA induces degeneration of nematode DAergic neurons. Day 6 wild type nematodes with DsRed-marked DAergic neurons were left untreated (control) or treated with 1 mM 6-OHDA alone or in combination with 1 mM imipramine. Details are described in Experimental procedures. Representative DsRed fluorescent (top panels) and bright field (D–F) images are shown with DAergic neurons located in the nematode head (CEPs and ADEs) indicated by arrows. (G–I) Quantification of 6-OHDA-induced DAergic neuron degeneration as a function of age in wild type and loss-of-function lrk-1 mutant (km17) nematodes. Wild type (g, i) and lrk-1 mutant (km17) L3 nematodes (h, i) were incubated in solutions containing either vehicle (control, red), 2 mM 6-OHDA (black) or 2 mM 6-OHDA+1 mM imipramine (green) for 1 hour. DAergic neuron somas of hermaphrodites were counted on the indicated days and normalized to the mean number of DAergic somas observed in wild type L4 nematode larvae (See details in Experimental Procedures). Data represent the mean ± SEM of four independent experiments. Each experiment employed 20–30 nematodes. **, p<0.01 and ***, p<0.005 by two-way ANOVA. (TIF) [file pone.0022354.s001.tif]

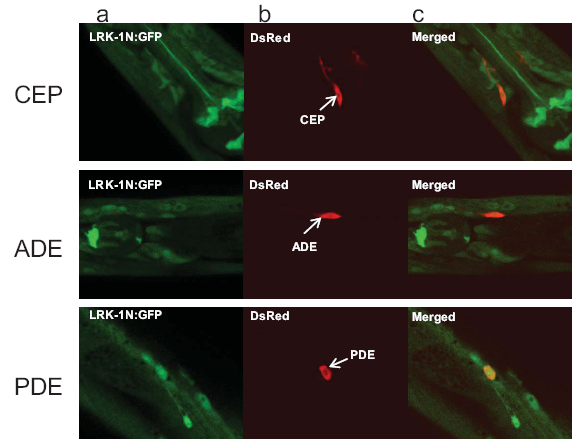

Supplement: Figure S2 — LRK-1 is expressed in nematode DAergic neurons. (a–c) Representative images of LRK-1N::GFP fusion protein (a), DsRed marking DAergic neurons (b), and merged images of a and b (c) demonstrate that LRK-1 is expressed in all three types of nematode DAergic neurons: CEPs (cephalic, upper panels), ADEs (anterior deirid, middle panels) and PDEs (posterior deirid, lower panels). Images show day 1 wild type nematode expressing LRK-1N::GFP and Pdat-1 driven DsRed. Living nematodes were immobilized with azide on agarose pads, and images were taken with a Leica cofocal microscope (See Experimental Procedures). (TIF) [file pone.0022354.s002.tif]

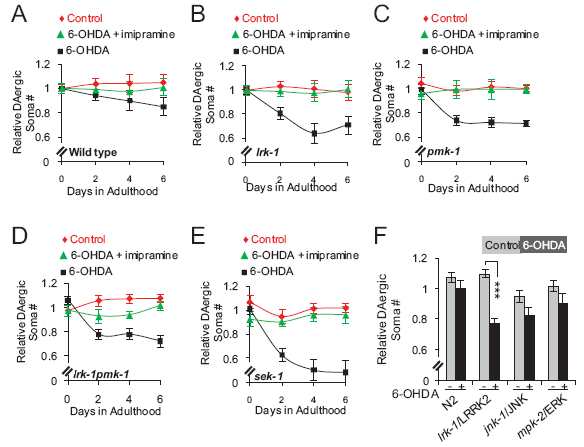

Supplement: Figure S3 — LRK-1 signals through p38 MAP kinase to protect DAergic neurons against 6-OHDA-induced neurotoxicity. (A–E) DAergic neuron degeneration is shown as a function of age in wild type (a), lrk-1/LRRK2 loss-of-function mutant (km17) (b), pmk-1/p38 null mutant (c), lrk-1(km17)pmk-1(null) double mutant (d) and sek-1/MKK6 null (e) nematodes incubated in solutions containing either control vehicle (red), 2 mM 6-OHDA (black) or 2 mM 6-OHDA+1 mM imipramine (green). Experimental details are provided in the Experimental Procedures section. Data represent means ± SEM of three independent experiments. Each experiment employed 20–30 nematodes. (F) Quantification of DAergic neuron degeneration in wild type, lrk-1 loss-of-function, jnk-1/JNK null, and mek-2/ERK null day 4 nematodes exposed to 2 mM 6-OHDA or control vehicle. Experimental details are provided in the Experimental Procedures section. Data represent means ± SEM of three independent experiments. Nematode numbers (n) varied from 10–30 in each experiment. ***, p<0.005; t-test. (TIF) [file pone.0022354.s003.tif]

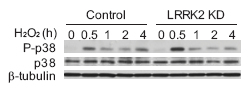

Supplement: Figure S4 — LRRK2 KD cells show similar activation of p38 under H2O2 exposure. Both MIX LRRK2 KD cells (LRRK2 KD) and SH-SY5Y vector control (control) cells were treated with 20 µM H2O2 for the indicated time. Western blot shows similar activation of p38 in both cell lines. (TIF) [file pone.0022354.s004.tif]

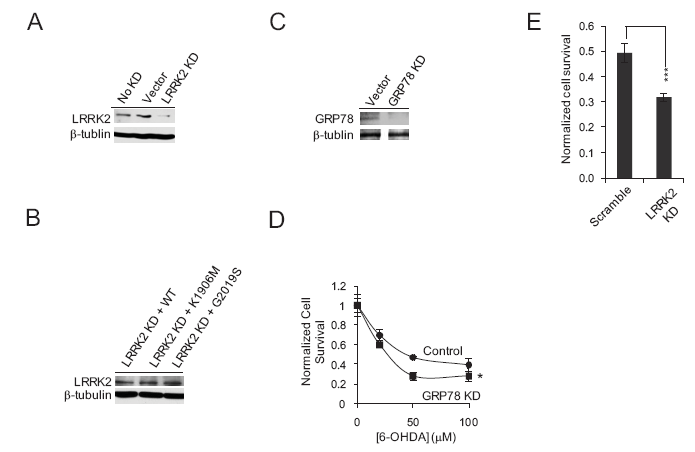

Supplement: Figure S5 — shRNA-mediated knock-down (KD) of LRRK2 and GRP78 expression in human SH-SY5Y cells. (A) Western blots show that 3′-UTR LRRK2 KD SH-SY5Y cells (LRRK2 KD) exhibited low levels of LRRK2 compared to SH-SY5Y cells without LRRK2 KD (No KD) or SH-SY5Y cells transfected with control vector (vector). (B) Western blots show that 3′-UTR LRRK2 KD SH-SY5Y cells transfected with WT (LRRK2 KD+WT), K1906M mutant (LRRK2 KD+K1906M) and G2019S mutant (LRRK2 KD+G2019S) LRRK2 evidenced similar levels of LRRK2. (C) Western blots show that SH-SY5Y cells transfected with a GRP78 shRNA set (GRP78 KD) exhibited less GRP78 expression than SH-SY5Y cells transfected with control vector (Vector). (D) Cells lacking GRP78 expression show increased sensitivity to 6-OHDA. Cell survival was assessed using an XTT-based calorimetric assay in SH-SY5Y cells transfected with a control vector (control, circles) and in MIX GRP78 KD SH-SY5Y cells (GRP78 KD, squares). Cells were exposed to the indicated concentrations of 6-OHDA for 24 hours prior to assessment of cell survival. Data are shown normalized to the level of survival in cell cultures not treated with 6-OHDA and represent the mean ± SEM of 3 independent experiments. *, p<0.05; two-way ANOVA. (E) Cells from a MIX LRRK2 KD SH-SY5Y line (LRRK2 KD) and SH-SY5Y cells transfected with a scramble shRNA (Scramble) were treated with 100 µM 6-OHDA for 12 hours. Cell viability was determined using an XTT-based calorimetric assay. Data represent the mean ± SEM of 3 independent experiments. ***, p<0.005 by t-test. (TIF) [file pone.0022354.s005.tif]

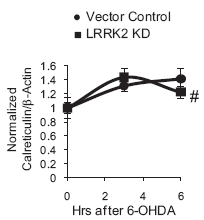

Supplement: Figure S6 — LRRK2 expression does not affect 6-OHDA-mediated induction of calreticulin transcription in SH-SY5Y cells. qPCR quantification of calreticulin induced by exposure to 100 µM 6-OHDA was compared between SH-SY5Y cells transfected with control vector (circles) and MIX LRRK2 KD (squares). X-axis shows periods of 6-OHDA exposure. Cells were exposed to 6-OHDA for specified periods and total RNA was extracted and transcribed into cDNA, followed by qPCR experiments. Details are provided in the Experimental Procedures section. Data represent the means ± SEM of 3 independent experiments. #, p>0.05 by two-way ANOVA. (TIF) [file pone.0022354.s006.tif]

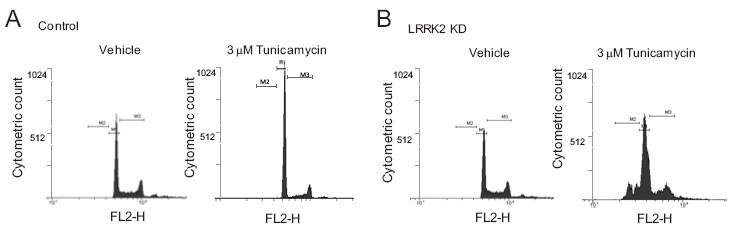

Supplement: Figure S7 — LRRK2 supports GRP78-mediated cell survival against tunicamycin. (A) LRRK2 KD cells are more vulnerable to tunicamycin-induced cell death than wild type cells. Sample flow cytometric traces of propidium iodide-stained control SH-SY5Y cells (Control) (A) or LRRK2 MIX KD SH-SY5Y cells (LRRK2 KD) (B) with (right panel) or without (left panel) 3 µM tunicamycin exposure for 16 hours. M1, M2 and M3 indicate cells with DNA content corresponding to the G0/G1 phase of the cell cycle, sub-G0 (apoptotic), and all other phases, respectively. FL2-H indicates the channel used to observe fluorescence. (TIF) [file pone.0022354.s007.tif]

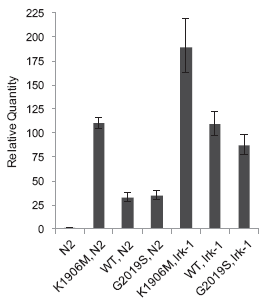

Supplement: Figure S8 — LRRK2 qRT-PCR confirmed the expression of WT LRRK2 and mutant forms K1906M and G2019S in wild type and lrk-1 mutant backgrounds. Wild type nematode lines and lrk-1 mutant nematode lines with pan-neuronal expression of WT LRRK2, K1906M or G2019S mutant LRRK2 (driven by the PH20 promoter) as well as DAergic neuron-specific expression of DsRed and command interneuron-specific expression of yellow fluorescent protein (YFP, driven by the nmr-1 promoter) were subjected to total RNA extraction, and qRT-PCR analysis. The relative quantity (RQ) value of WT LRRK2, K1906M or G2019S mutant LRRK2 vs. actin-1 were calculated. The error bar was based on the RQMin/Max confidence level that represents the standard error of the mean expression level (RQ value). (TIF) [file pone.0022354.s008.tif]
